# Microfluidic Sample Compartmentalization for Biomolecular Concentration Quantification Using a Nanopore Sensor

**DOI:** 10.1002/smtd.70924

**Published:** 2026-08-11

**Authors:** Morteza Safari, Juliana Chawich, Ali Najafi Sohi, Martin Charron, Vincent Tabard‐Cossa, Michel Godin

**Affiliations:** ^1^ Ottawa‐Carleton Institute for Biomedical Engineering Ottawa Ontario Canada; ^2^ Department of Physics University of Ottawa Ottawa Ontario Canada

**Keywords:** biological system, biomolecule, calibration, detection limit, microfluidics, nanopore, nanotechnology

## Abstract

Determination of the concentration of a biomolecule in a sample is a fundamental measurement in analytical chemistry and central to many diagnostic applications. Solid‐state nanopores (ssNPs) stand out as leading candidates to quantify biomolecules due to their capacity to achieve label‐free electrical detection with single‐molecule resolution. Often, the concentration of a biomolecular sample is estimated in relation to the ssNP capture rate, that is, the number of molecules translocated by the nanopore per unit time. However, this method poses challenges due to variability in capture rates, which have proven difficult to control experimentally. In this work, we introduce a microtrap, that is, a microfluidic device with an integrated nanopore sensor, that leverages sample compartmentalization in known pL volumes allowing direct molecule counting and eliminating the need for capture rate calibration. We demonstrate direct quantification of pico‐ to nanomolar concentrations of double‐stranded DNA (dsDNA) and green fluorescent protein (GFP) molecules in minutes and without the need for calibration of the nanopore sensor capture rate. Moreover, we demonstrate the ability to preconcentrate the biomolecular sample by real‐time tuning of the microtrap volume, which can be used to reduce the measurement time and to lower the detection limit.

## Graphical abstract

We compartmentalize samples into picoliter volumes using a microfluidic device, allowing us to determine biomolecular concentration by counting molecules to depletion within a known volume without calibration. By integrating a nanopore sensor for this counting process, we also obtain real‐time data on the size, shape, and conformation of each individual molecule.

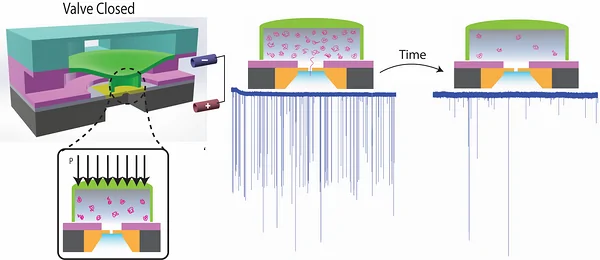

## 1 Introduction

Quantification of the concentration of biomolecules, ranging from nucleic acid to proteins, is one of the primary steps of many important assays in biochemistry, biology, environment, food, public health, and medicine [1, 2, 3, 4]. Nanopore‐based biomarker quantification offers a significant benefit by providing single‐molecule detection resolution in label‐free, miniaturized and portable formats [5, 6, 7], enabling the direct and precise quantification of individual biomolecules, which not only enhances diagnostic accuracy, but also provides a better picture of the heterogeneity of diseases at a molecular basis [8]. To this end, nanopore technology has attracted significant attention following the success of the commercial Oxford Nanopore sequencing platform, which enables single‐molecule, long‐read DNA sequencing [9]. While biological nanopores generally exhibit lower noise levels and higher sensitivity for molecular sensing, solid‐state nanopores (ssNPs) are promising candidates for high‐throughput quantification of charged molecules. This advantage arises from their tunable pore size, mechanical robustness, and compatibility with integration into other analytical tools, such as microfluidic platforms [10, 11]. ssNP devices feature a single nanoscale hole in a thin (∼10 nm) electrically insulating membrane. When an external force is applied across a nanopore linking two electrolyte‐filled chambers, molecules (e.g., DNA, proteins, etc.) are captured and pass through the pore one at a time. In the presence of a bias voltage, molecular passage creates a transient blockade in ionic current that is commensurate with molecular size, shape, and conformational state [12, 13]. Such external forces can include electrophoretic forces generated by applying a transmembrane voltage [14, 15], diffusio‐phoretic forces resulting from a concentration gradient across the nanopore [7, 16, 17], thermophoresis achieved by exposing the nanopore to temperature gradient [18], hydrodynamic drag forces induced by either electroosmotic flow (EOF) [19, 20] or pressure driven flow [21, 22, 23]. The latter is especially meaningful for capturing neutral or more weakly charged molecules than DNA [22, 24].

While nanopores offer remarkable sensitivity, reliably quantifying the concentration of biomolecules remains challenging due to uncertainties in the methods used for measuring concentration with nanopore sensors [25]. Currently, the most commonly used method for quantifying target concentration with a nanopore is by calibrating it relative to its capture rate (i.e., # of molecule detected per unit time). In this approach, the nanopore is first calibrated using a sample of known concentration to measure its capture rate. The unknown sample is then loaded, and the ratio of capture rates is used to determine its concentration, given the linear relationship between capture rate and analyte concentration [25]. However, even when fabricated with sub‐nanometer precision, ssNPs exhibit significant pore‐to‐pore variability in their capture characteristics and transport properties, induced by minute geometric and surface charge variations which are not currently controllable, making generalization of results between nanopores a significant analysis challenge [25]. Apart from pore‐to‐pore variation, it has been shown that the capture rate of a nanopore can fluctuate during an experiment, necessitating a real‐time calibration of the nanopore capture rate. While some sources of these fluctuations, such as initial pore size, salt concentration, applied voltage, and membrane thickness, can be controlled with reasonable precision, other factors cannot. These include changes in pore size during the experiment, variations in effective pore height, and differences in pore geometry. Because these parameters are neither easily controlled nor directly measurable, their contributions to nanopore variability cannot be quantified. Consequently, the overall source of variability in nanopore measurements remains uncertain, posing a critical challenge that limits the reliability of nanopores for concentration measurements [25, 26, 27, 28]. To this end, introducing another biomolecule with a known concentration was proposed to act as an internal calibrator, and account for all sources of variability that can interfere with the quantification experiment [25]. Nonetheless, this introduces additional complexities to the operation, as the concentration, length, shape, and capture regime of the calibrator molecules must be carefully selected to ensure that their electrical signature can be distinguished from that of the target molecules.

Another challenge is at low concentrations (<1 nM), where ssNP‐based methods can be time‐consuming due to their low capture rate, typically around 1 Hz per nM [7]. This is mainly due to the mechanism of molecule transport to the nanopore, which is dictated by a balance of diffusion and electrophoresis [29]. Different methods have been used to enhance the capture rate, including exposing the nanopore to asymmetric salt conditions [7], or employing tangential flow over the nanopore [30]. Both cases result in enlarged capture volumes, defined as the hemispherical volume where electrophoretic forces dominate over diffusion, enabling efficient motion toward the pore leading to molecule capture. In asymmetric salt conditions, the capture volume and capture rate increase due to the redistribution and amplification of the electric field on one side of the nanopore [7, 17]. While the tangential flow approach does not alter the electric field, it enhances the capture rate through a combination of diffusion and convection driven transport of molecules and has resulted in a five‐fold increased capture rate [30]. Another approach to enhance the capture rate is to locally increase the concentration of the biomolecule sample over the nanopore using dielectrophoresis. In this vein, coating gold on the surface of a nanopipette has shown to boost the capture rate by a factor of 80 [31]. In another report, functionalized magnetic microbeads with target analytes attached were optically trapped near the nanopore, and the analytes were then released by controlling the temperature momentarily increasing the capture rate by 80‐fold [32].

In recent years, the integration of nanopore sensors within microfluidic networks has gained significant attention, leveraging the best aspects of both platforms. In particular, microfluidics provide pL‐range liquid control and handling through actuation of on‐chip microvalves of value for biosensing applications. Here, we present a microfluidic platform with an integrated nanopore sensor capable of accurately quantifying biomolecular concentration, while offering the ability to pre‐concentrate the sample. The proposed design involves an on‐chip valve that can be actuated to lower a microwell over the nanopore. This microwell, hereafter referred to as ‘microtrap’, confines the sample within a cavity of a known volume (pL‐range). Then, using the resistive pulse technique, the embedded nanopore is used to count biomolecules contained within the microtrap to depletion, thereby allowing the inference of the sample concentration. With this approach, concentration quantification is achieved without requiring the calibration of the nanopore sensor's capture rate, simplifying the quantification process. We further show that the microtrap can be used to locally concentrate the sample over the nanopore, thus reducing the overall counting time, and allowing quantification of low concentration samples. Finally, we demonstrate the device's ability to measure protein concentration by trapping, sensing and depleting GFP protein using the embedded nanopore sensor.

## 2 Result and Discussions

### 2.1 Microfluidic Device Design

**FIGURE 1:**
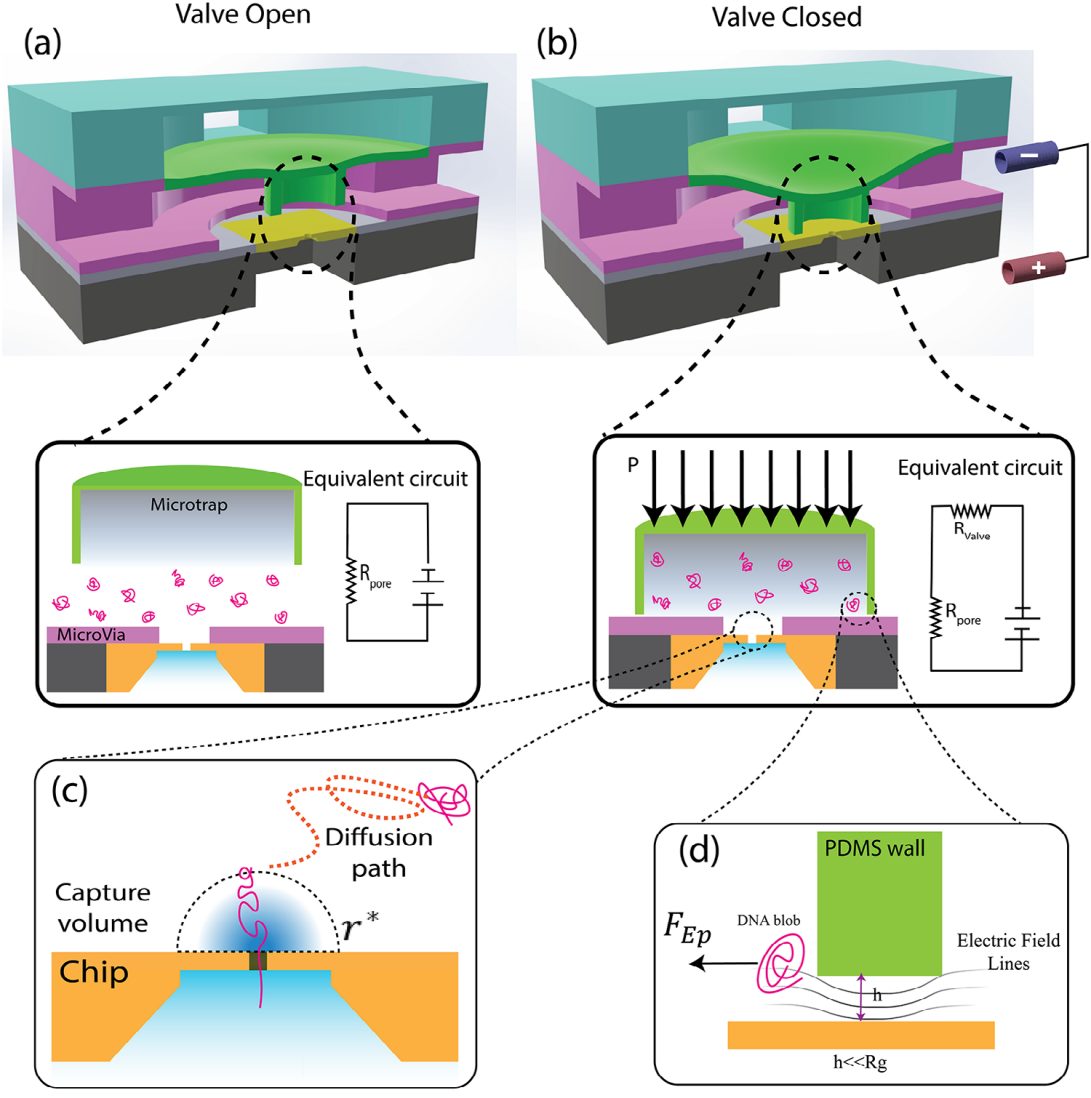
Conceptual design of the nanopore‐microfluidic device for concentration quantification. (a) A molecular sample can be introduced into a microchannel (purple) located above the nanopore. Here, the microtrap located above the nanopore is open. (b) Closure of the microfluidic valve creates a small compartment around the nanopore, the hydrophilicity of surfaces helps maintain a very thin film (on the order of nm) of solution between the PDMS wall and chip surface (referred to as the “gap”). (c) Schematic depicting the nanopore electrophoretic capture process. (d) Schematic representation of the DNA blob trapped at the microtrap edges. The schematics are not drawn to scale. Rg represents the characteristic radius of gyration of dsDNA confined within the microtrap.

ssNPs were made in thin (10–20 nm) commercially available silicon nitride membranes, supported by a thicker silicon frame (5 × 5 mm; 200 µm thick). This silicon chip was embedded within a Polydimethylsiloxane (PDMS) structure with microchannels located above and below the nitride membrane. To enable the compartmentalization of molecular samples nearby the nanopore sensor, a microtrap feature was molded in the upper microchannel (Figure 1). The pressure in an adjacent microchannel can be controlled to lower this microtrap over the nanopore sensor to compartmentalize an aliquot of the biomolecular sample. Figure 1a,b illustrates the two operational modes of the microfluidic device integrating a nanopore sensor. In the first mode, the microtrap is left open. Here, the biomolecular sample is thus contained in a channel acting as a reservoir, whereby the translocation of molecules through the nanopore does not alter the effective molecular concentration on the *cis* side of the nanopore (Figure 1a). In the second mode, the microvalve is pressurized and the microtrap is lowered to softly contact the membrane surface, creating an entropic trap over the nanopore that confines a finite number of molecules (Figure 1b). While the molecules cannot escape the microtrap due to their size (Figure 1b and 1d), the microtrap leaves behind a thin layer of electrolyte, which makes the nanopore electrically accessible. The existence of a thin layer of electrolyte that allows ionic current to flow has been previously established by our group [33]. When a DC voltage is applied to an electrode located within the microchannel but outside the microtrap, the entrapped molecules are counted to depletion using resistive pulse technique (Figure 1c), allowing precise determination of concentrations. The trap when closed also focuses the electric field lines from the far away electrodes thus adding an electrophoretic barrier acting to further prevent molecular escape (Figure 1d).

### 2.2 Counting Molecules From a Finite Volume

Conventionally, sample concentration is estimated from the nanopore capture rate, J, which is linearly proportional to concentration. In practice, J is obtained as the slope of the cumulative molecule count versus time. Therefore, the concentration of an unknown sample can be determined by measuring its capture rate and comparing it with that of a calibrator sample of known concentration. However, as Charron et al. have shown, this calibration curve varies due to intrapore and interpore variability, necessitating real‐time calibration [25]. In this study, we also demonstrated both inter‐ and intrapore variability in nanopore capture rates by testing the pores in conventional fluidic cells described in our previous work [34] which is equivalent to open valve mode. As shown in Figure S1a, performing two tests with the same sample (1 nM of 2 kbp dsDNA) on two different nanopores, indicated by blue and dark red symbols, revealed distinct capture rates, represented by the slopes of the fitted lines. This observation highlights the need for calibration of each nanopore due to capture rate dependency on pore geometry which varies from pore to pore. However, even after calibrating a nanopore with a known concentration (1 nM of 2 kbp dsDNA), the measured concentrations still deviated significantly from the expected values (Figure S1a,b). This discrepancy arises from changes in the normalized capture rate after calibration, illustrating intrapore variability. Consequently, measured concentrations varied, with deviations of ∼30% commonly reported [25] and occasionally reaching up to 150% for a 50 pM sample, despite all measurements being conducted under similar operational conditions (400 mV bias). It must be noted that the reported errors do not account for uncertainties in curve fitting, which can be significant when the cumulative molecule counts versus time deviates from linearity, due to intrapore variability, as observed for the 50 pM concentrations (green) in Figure S1a. These limitations motivated our transition from conventional capture rate‐based measurements to direct counting of molecules in a finite volume with no calibration step required.

**FIGURE 2:**
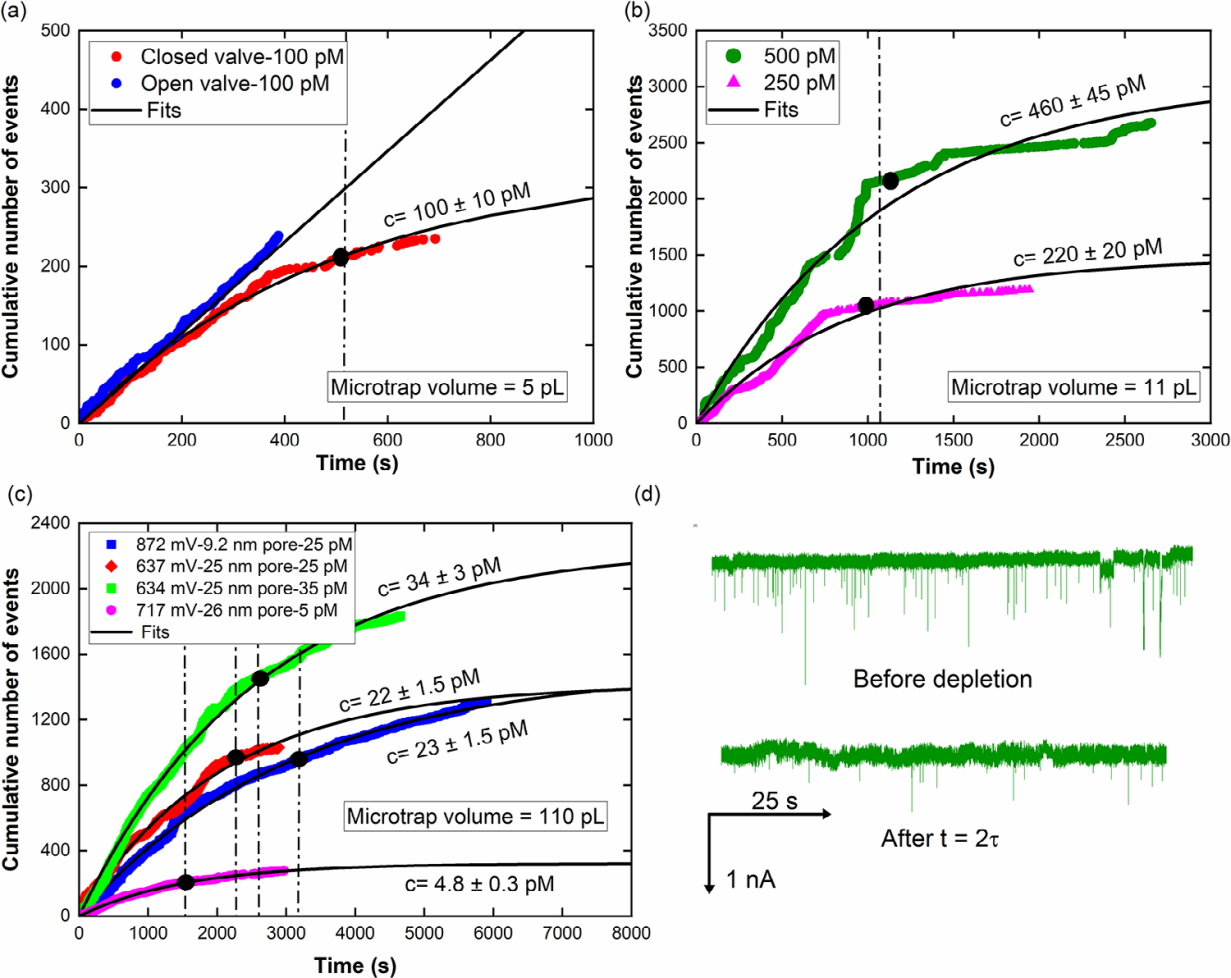
(a) Cumulative single molecule counts as a function of time for 100 pM of 2 kbp dsDNA in open and closed valve (P_valve_ = 1 psi). Experiments performed within 5 ± 0.5 pL microtrap volume using a nanopore with diameter and length of 8 ± 0.5 nm and 20 ± 2 nm, respectively, under an applied bias voltage for open and closed valve of 900 and 950 mV respectively. (b) Cumulative single molecule counts as a function of time for 500 pM and 250 pM of 2 kbp dsDNA samples along with the respective fitted curves. Experiments were performed within a 11 ± 1 pL microtrap volume and at 437 mV bias voltage using a nanopore with diameter and length of 17.6 nm and 20 nm, respectively. (c) Cumulative number of events as a function of time for 25 pM of 2 kbp dsDNA in closed valve (P_valve_ = 2 psi) at two different experimental condition and with two different nanopore, and 35 pM of 2 kbp dsDNA in closed valve (P_valve_ = 2 psi). (d) Representative ionic current traces for the 500 pM data in (d) at the beginning and the end of the experiment. All experiments were performed in 3.6 M LiCl pH8. The black circles in a–d is representing the time to response that has been calculated through curve fitting. The R^2^ for the fit in panel (a) was 0.99. In panel (b), the R^2^ values were 0.97 and 0.96 for the 500 pM and 250 pM samples, respectively, and 0.99 for all other fits. The c annotated on figures indicate the measured concentration.

We start by discussing and comparing the kinetics of nanopore capture with the open and closed microtrap in order to establish the method for concentration quantification with our microdevice. The kinetics of biomolecular capture by the nanopore was measured by introducing 2 kbp dsDNA at 100 pM into the microchannel on the upstream (*cis*) side of the nanopore. When the microtrap was kept open (open valve) and a bias voltage of –900 mV was applied on the *cis* side electrode, molecular translocation events were detected. The cumulative number of events as a function of time is displayed (blue data points) in Figure 2a. As can be seen, the data follow a relatively linear trend indicating that the mean capture rate remained constant which is expected since the dsDNA concentration in the large reservoir of the microchannel can be considered to remain constant during the experiment (only a few hundreds of molecules are translocated in a reservoir containing > 10^6^ molecules).

When the microtrap was closed by applying a pressure of 1 psi to the control valve microchannel, the voltage was adjusted to −950 mV to ensure that the ionic current open baseline matched that of the open microtrap value, thus leaving the capture kinetics unchanged. As can be seen in Figure 2a, the slope in the cumulative number of events versus time slowly decreases as more molecules are translocated. The gradual slope reduction highlights a gradual reduction in capture rate, which in turn indicates a reduction in dsDNA concentration inside the microtrap, since capture rate is correlated with concentration. This is expected since when the microtrap is closed, the molecular concentration is expected to reduce as molecules translocate through the nanopore and are depleted from the microtrap volume. Under closed‐valve conditions, it becomes possible to count the molecules confined within the microtrap to complete depletion, which happens when the red data points in Figure 2a reaches a plateau indicating that the capture rate approaches zero. In this case, the concentration can be determined by dividing the total number of counted molecules by the known volume of the microtrap. Therefore, when using microtrap for quantification the pore size, length, geometry, surface properties, and other sources of variability do not affect the measured concentration.

Ideally, the microtrap quantification experiment should continue until no more events are detected by the nanopore, that is, complete depletion. However, this can be time‐consuming and especially so for the larger microtrap volumes, given that molecules need to diffuse to the capture volume to be counted. Therefore, modeling the capture kinetics to predict the asymptotic value for number of captured molecules, N_0_, can significantly reduce the runtime for quantification. Assuming the concentration within the microtrap is uniform, the number of molecules within the microtrap at time t, N(t), can be modeled by noting that the capture rate *J* is defined as *J* = −*dN*/*dt* and assuming a linear dependence on local microtrap concentration, *J* = *Rc*(*t*) = *RN*(*t*)/*V*, where *R* is the normalized capture rate and *V* is the microtrap volume. Equating both capture rate expressions, and noting that the cumulative number of captured events is N_cap_ (t) = N_0_ − N(t) we find the following expression for N_cap_(t):

(1) $$N_{\mathrm{cap}}\left(t\right)=N_{0}\left(1-\exp\left(-t/\tau \right)\right),$$

where the constant τ is the time‐to‐response (TTR) constant defined as $\tau =\frac{V}{R}\;,$ and corresponds to the time when 63% of the total molecules are counted. It must be noted that the normalized capture rate, R, is not necessarily constant in the experiment, leading to deviations of the experimental data from Equation 1. Such deviations from the model may introduce errors in the measured concentration, which will be further elaborated in the following sections.

Equation (1) can be used to predict the number of molecules confined within a microtrap of known volume to define the sample concentration. In Figure 2a, a DNA sample at a concentration of 100 pM was confined within a microtrap of volume 5 ± 0.5 pL. A fit to the experimental data using Equation 1 yields a measurement of N_0_ = 330 ± 5 confined molecules, from which the concentration of the sample is determined, c = N_0_/(N_A_V), where N_A_ is Avogadro's number) to be 100 ± 10 pM, agreeing well with the prepared sample concentration of 100 pM. It must be noted that the uncertainty in the measured concentration is calculated using error propagation principles, encapsulating all significant sources of errors. This includes uncertainties arising from intrapore normalized capture rate variability and uncertainty in the determination of the microtrap volume. The details of the calculation of the uncertainty are reported in Section S2 of the Supplementary Information. It is important to note that if this experiment were repeated several times, additional uncertainty could appear due to the random error introduced by the microtrap sampling process. Specifically, in each experiment, the microtrap randomly samples a subset of DNA molecules from the bulk solution, which may contain a slightly higher or lower number of molecules according to Poisson statistics. Throughout the manuscript, all reported N_0_ values ascertained from curve fitting were rounded according to their corresponding uncertainties. However, for concentration calculation we have used unrounded N_0_ values, and the resulting concentrations were then rounded based on their associated uncertainties.

To build on these findings and further validate the concept of counting to depletion, another set of experiments was conducted using a microtrap with approximately twice the volume (11 ± 1 pL) for DNA samples with concentrations of 500 pM (green) and 250 pM (pink), as shown in Figure 2b. In this case, 3070 ± 20 molecules and 1480 ± 20 molecules were measured, corresponding to concentrations of 460 ± 45 pM, 220 ± 20 pM, respectively. These results (Figure 2b) agree with the expected concentrations of 500 pM and 250 pM given a typical pipetting uncertainty of 10%.

In another set of experiments, we employed microtraps with relatively larger volumes, 110 pL, to highlight the robustness of the technique. As shown in Figure 2c, two independent tests were conducted under entirely different operational conditions (pore size and voltage), but using the same input concentration, yielding nearly identical measured concentrations. Specifically, the measured concentrations in these tests were 23 ± 1.5 pM and 22 ± 1.5 pM, both in good agreement with the original sample concentration of 25 pM. This demonstrates the robustness of the approach and its insensitivity to pore‐to‐pore or device‐to‐device variations. Furthermore, a separate experiment using a 35 pM sample resulted in a measured concentration of 34 ± 2 pM, further showcasing the accuracy and reliability of this platform (see Figure 2c). Using a different device and nanopore, the concentration of 5 pM was also tested which resulted in measuring 322 ± 2 molecules which translate to 4.8 ± 0.3 pM concentration (see Figure 2c).

One concern when using nanopores, particularly larger pores (∼25 nm) to detect relatively small molecules such as 2 kbp dsDNA, is the potential loss of events due to the limited temporal resolution of the electronics. Missed events would bias concentration measurements, as our method relies on detecting and accounting for all molecules present in the microtrap. To ensure that the fraction of undetected events is negligible, we employed a first passage time (FPT) model [35] to estimate the percentage of translocation events occurring below the temporal cutoff, ∼10 µs, required for fully resolved detection. This analysis shows that the fraction of missed events is insignificant, below 2%, for both the 25 pM and 35 pM experiments presented in Figure 2c (red and green curves, respectively). For additional details on the fraction of missed events and its estimation using the FPT model, see Section S3 of the Supplementary Information.

### 2.3 Kinetics of Counting: TTR

We now address the TTR of the microtrap depletion counting process, that is, the τ variable in Equation 1, which encloses information about the capture kinetics. According to the simple model introduced above (Equation 1), the TTR is independent of sample concentration. At first glance, this might seem counterintuitive, as higher concentrations are typically associated with increased capture rate, but more molecules need to be counted to reach the same relative depletion level, ultimately leading to identical TTRs independent of sample concentration. This was verified by running two experiments at two different sample concentrations (500 pM and 250 pM) under identical voltages (437 mV) and in the same nanopore (17 nm diameter) in a V = 11 ± 1 pL microtrap (Figure 2b). In both cases, the time constants, τ, for both fits were 1100 ± 10 s and 900 ± 20 s, respectively. The slight discrepancy between these values can be attributed to variations in the normalized capture rate of the nanopore, R, which in turn influence the measured TTR (τ) across different experiments.

As τ = V/R, the TTR can be shortened by either reducing the microtrap volume and/or increasing the normalized capture rate (R) which, for example, can be increased by applying higher voltages or using larger pore sizes. In the experiment of Figure 2a, although a smaller pore size is used, 8 nm, the smaller microtrap volume (V = 5 ± 0.5 pL) and higher voltage (950 mV) yielded a shorter TTR with τ = 500 ± 12 s. Increasing the microtrap volume to 110 pL was found to significantly increase the TTR, since more molecules need to be counted for determining the concentration. In an experiment conducted at 25 pM using a 9.2 nm pore and an 872 mV bias, the measured τ was 3060 ± 20 s. We note that the use of different applied bias voltages for experimental results presented in Figure 2 is mainly due to two reasons. First, it is important to note that the resistance induced by valve closure can vary between experiments, due to geometrical differences and device to device variability. Consequently, since our goal is, for a given pore device, to maintain the same current baseline as the open valve state, the adjusted voltage may differ from one device to another. Second, the applied voltage was carefully adjusted to balance operational considerations, including nanopore stability, the ability to resolve individual molecules, and achieving short TTR.

In contrast, for the same concentration of 25 pM but with a 25 nm pore and a lower bias of 637 mV, τ was reduced to 2050 ± 30 s. This highlights that the experiment can be tuned to influence the TTR. Using larger nanopore diameters is particularly effective in reducing the TTR, as the capture rate, defined as the radius of the hemispherical capture volume, scales with the square of the pore diameter. However, this capture rate is only linearly correlated with the applied bias voltage [30]. We note that the 25 nm nanopore used in the experiment shown as the red curve in Figure 2d was also used to measure the concentration of a 35 pM sample, resulting in a TTR of 2570 ± 15 s. As previously discussed, TTR is independent of sample concentration; thus, the observed difference in TTR between experiments is attributed to differences in normalized capture rates. In this vein, for the 5 pM test, the measured TTR was 1540 ± 20 s, which is significantly shorter than those of the other tests presented in Figure 2c, all of which were conducted in the same volume, 110 pL, but with different devices. This difference is mainly due to the use of different operational conditions and nanopores, which did not affect the measured concentration.

We next address the question of reliable curve fitting, namely the impact of the experiment duration on the precision of the concentration measurements. Naturally, fitted data from experiments that run longer result in more precise values of N_o_ than shorter ones. This is elaborated in Section S4 of Supplementary Information, which further suggests that experimental times can be reduced, but should be longer than 1 × τ to optimize statistical precision and given the variability in the normalized intrapore capture rate throughout the experiment. For reference, the experiment highlighted in Figure 2a (100 pM) had a runtime of 1.4 τ, and the experiments in Figure 2b (500 pM and 250 pM) were run for 2.3 τ and 2 τ, respectively. Additionally, for the 110 pL microtrap, the 35 pM test was conducted for 1.7 τ, while the 25 pM test represented by the red curve in Figure 2c was run for 1.2 τ. In contrast, the other 25 pM (blue curve in Figure 2c) and the 5 pM tests were both performed for 2 τ. In all cases, the measured concentrations were consistent with the expected values.

While the proposed model for measuring sample concentration via curve fitting is robust against interpore variability, significant intrapore variability can still impact the accuracy of the results (see Figure 2b). Although such intrapore variability is not frequently observed, when it does occur, it results in higher measurement uncertainty. This is evident in Figure 2b, where the measured concentration closely aligns with the expected value, yet the fitting uncertainty remains high. This increased uncertainty arises from variations in the normalized capture rate during the experiment, which lead to deviation of experimental data from the model suggested for describing the kinetics of microtrap depletion. Earlier we established that counting to full depletion can require long measurement times, and without prior knowledge of the concentration, it is impossible to determine when the microtrap is fully depleted. However, by monitoring the capture rate in real time, it is possible to infer the fraction of molecules captured by the nanopore. In this approach, counting more than 95% of the trapped molecules can still yield a reliable concentration measurement without the need for curve fitting. In this vein, when the capture rate drops to 1%–5% of its initial value, it indicates 95%–99% depletion, at which point the number of counted molecules can be taken as representative of the sample concentration within the microtrap. Although this method was not implemented in the present study, one potential approach to achieve concentration measurements with lower uncertainty is to perform multiple experiments using the same sample in parallel. Such parallelization can reduce measurement uncertainty stemming from the curve fitting.

### 2.4 Scaling of TTR

**FIGURE 3:**
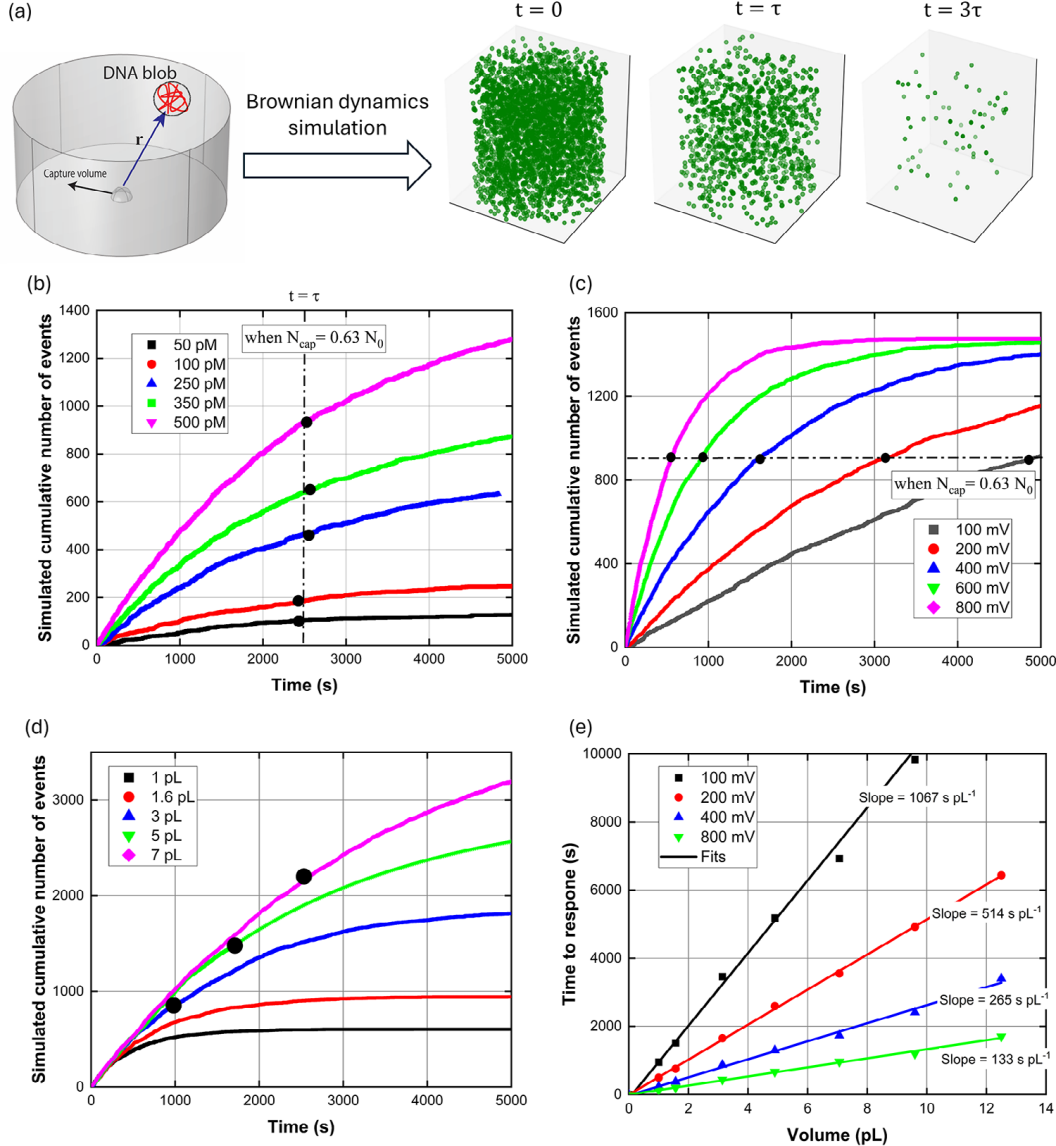
(a) Schematic of simulation domain (left panel, not to scale) and example of simulated particles that has been captured by nanopore's capture volume at different time point. (b) Simulated cumulative number of captured particles over time, modeled for a microtrap with a volume of 5 pL at five different molecular sample concentrations, showing that the TTR (marked by a black dot on the curves) is independent of the sample concentration. (c) Effect of the bias voltage on the TTR at a set concentration of 500 pM. (d) Effect of the microtrap volume on the TTR at set 1 nM concentration and 400 mV bias voltage. (e) Combined effect of volume and voltage on the TTR, which shows linear relationship between the TTR and microtrap volume. The slopes obtained based on a linear curve fitting were used to estimate the TTR (defined as the time that 63% of the total molecules were captured and represented by black dots) for any microtrap volume. All the simulations were performed for a nanopore with diameter of 15 nm and a length of 20 nm, under an applied bias voltage of 400 mV, unless stated otherwise.

While TTR, τ, is a crucial factor in determining the duration of quantification experiments for accurate curve fitting, its determination presents a significant challenge. This is because TTR does not directly reveal how long the experiment needs to run; it can only be calculated retroactively through curve fitting, after running the experiment. Studying the parameters affecting TTR is further complicated by the inherent pore‐to‐pore variability, making systematic experimental studies difficult and comparisons challenging. Hence, Brownian dynamics simulations were undertaken to study the effects of different geometrical and operational parameters that affect the TTR. In this venue, a simplified version of the diffusion‐limited capture regime of dsDNA molecules was used to model and determine the time scale associated with counting to depletion in a fixed microtrap volume. To this end, dsDNA was modeled as points with predetermined diffusion coefficient, and their motion within a cylindrical microtrap, and outside of nanopore capture volume was described using Brownian motion [30]. The simulation focused on tracking the position of spherical blobs diffusing inside the microtrap until they randomly diffused inside the capture volume, at which point they were assumed to be instantly captured by the pore (Figure 3a). Therefore, in this model, the DNA was not simulated within the capture volume or inside the nanopore, where it undergoes non‐equilibrium deformation and its conformation changes in a complex manner [17]. Further details about the simulation are provided in the Supplementary Information Section S5.

Figure 3b shows cumulative number of capture events vs time obtained for different concentrations in a 5pL microtrap, and as can be seen the simulation data confirms that the TTR is not dependent on sample concentration. Moreover, the effect of voltage on the TTR was studied by varying the capture volume accordingly throughout simulations. Figure 3c shows decreased TTR values for larger bias voltages, Δϕ, leading to a faster response. This trend is expected because the characteristic response time scales inversely with the capture rate, that is, ∼ R^−1^. In the diffusion‐limited capture regime, the normalized capture rate, R, can be expressed as regime R = 2πDr*, with D and r* are respectively being diffusion coefficient and radius of nanopore capture volume. Since the capture radius linearly increases with the applied bias voltage, as described by Equation S9, the response time decreases with increasing applied voltage, *τ ∼ Δ* $\phi^{-1}$ consistent with our simulation results Besides, since the normalized capture rate, R, is related to the pore's diameter, d, and length, L, pore geometry is expected to impact TTR. Based on the expression for R and Equation S9, scaling relationship between the TTR and the nanopore geometry can be expressed as τ ∼ d^−2^ and τ ∼ L. Therefore, increasing the nanopore diameter and decreasing the nanopore length are both expected to reduce the TTR and provide a faster device response. Physically, a larger nanopore diameter enhances the molecular capture rate by increasing the effective capture volume, while a shorter nanopore reduces the electrical resistance of the pore and increases the electric field strength for a given applied voltage, thereby accelerating molecular transport toward the nanopore.

The diffusion simulations also demonstrate that the TTR is proportional to the microtrap volume, which is expected, as a larger microtrap volume contains a greater number of molecules at the same concentration (Figure 3d). While smaller microtrap volumes provide faster response, the use of standard microfabrication processes sets a lower limit of the microtrap dimensions. Moreover, it must be noted that, when curve fitting is used for measuring the concentration of a sample, the initial number of molecules compartmentalized within the microtrap should be large enough to render a reliable curve fitting. Figure 3e summarizes the dependence of TTR on microtrap volume and applied bias voltage.

### 2.5 In Situ Preconcentration and Increase in Capture Rate

**FIGURE 4:**
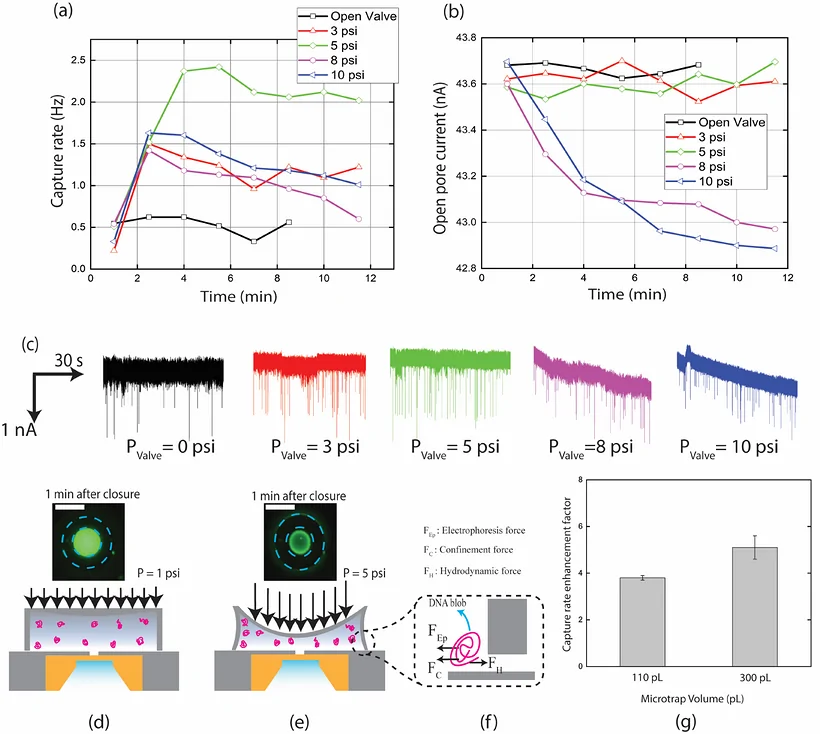
(a) Change in capture rate and (b) Open pore current over time, at varying valve pressures for a microtrap with 110 ± 7 pL volume. (c) Representative ionic current traces 3 min after valve closure for 500 pM of 2 kbp dsDNA under a 200 mV bias voltage in a 14 ± 0.5 nm pore within a 12 ± 2 nm‐thick SiN membrane, shown under different applied pressures on the microtrap. (d) Fluorescent image of microtrap filled with fluorescein in the top panel and schematic representation of the microtrap at bottom panel after pressurization to a moderate pressure level that does not induce trap deformation. (e) Fluorescent image of microtrap filled with fluorescein in the top panel and schematic representation of the microtrap at bottom panel after pressurization to a high‐pressure level that causes deformation that leads to decreasing the fluorescent intensity in the microtrap, since fluorescein molecules are small, and can escape from the microtrap with outward flow. (f) Schematic representation of forces applied on the DNA blobs in the vicinity of the microtrap wall, where electrophoretic and confinement force apply in the opposite direction of the hydrodynamic mass transport induced by the outward flow. (g) Capture rate enhancement factor at 5 psi valve pressure for microtrap volumes of 110 ± 7 pL and 300 ± 10 pL, illustrating the effect of the trap volume on the capture rate enhancement. For the 300 ± 10 pL microtrap, experiments were conducted with a 200 mV bias across a nanopore with an 11 nm diameter and 12 nm length. All experiments used a 500 pM solution of 2 kbp dsDNA, and the thickness of the microtrap PDMS layer was 20 µm. The scale bars in d) and e) are both 80 µm. The error bars in g) represent the standard deviation from 3 experiments.

Upon valve closure (lowering the microtrap over the nanopore), the initial capture rate was found to be the same as under open valve conditions, which is evident from the similar N_cap_ vs *t* slopes observed in both open and closed states in Figure 2a. This shows that the initial molecular concentration inside the microtrap was the same as it is in the rest of the microchannel and that the microtrap did not affect the nanopore's performance. However, when the valve pressure is set to pressures greater than 1 psi (the minimum pressure for closure, as shown in Section S6 in Supplementary Information), the nanopore's capture rate is affected. Figure 4a shows that under valve pressures between 1 psi and 5 psi, the capture rate increased significantly compared to the open valve case (0 psi). Additionally, when the valve pressure exceeded 5 psi, the ionic current baseline was observed to decrease over time, as shown in Figure 4b,c. It must be noted these experiments were performed within microtraps with volumes of 110 ± 7 pL. Using this larger volume, exponential decrease in capture rate due to depletion does not occur until after ∼2–3 h, based on operational condition used (14 ±0.5 nm pore with 200 mV applied bias voltage). Therefore, any change in capture rate over first 10 min is not due to the depletion effect. To explore this phenomenon using fluorescence microscopy, the microtrap was used to trap fluorescein, a small fluorescent molecule, in the absence of any bias voltage. As shown in Figure 4d, when the microvalve was pressurized to 1 psi, the intensity of fluorescence remained constant over time and uniform across the microtrap. In contrast, when applying 5 psi to the valve, the fluorescein intensity gradually decreased over the course of one minute (Figure 4e). We attribute these observations to the fact that under high valve pressure (>1 psi), the PDMS microtrap deforms, causing a build‐up of pressure within the microtrap that contains incompressible fluid. This induces convective flow of both electrolyte and small fluorescein molecules outward, primarily along the edges of the microtrap (see Figure 4d,e), resulting in decreasing fluorescent intensity over time (see the top panel of Figure 4e).

When trapping larger DNA instead of fluorescein molecules, DNA sample concentration within the microtrap gradually increases as the solution is squeezed out of the trap volume, while the DNA molecules remain sterically trapped. This is a reasonable assumption for three reasons. First, the size of the DNA blobs, R_g_ ~ 100 nm, is significantly larger than the microtrap gap height, estimated to be ∼0.5 nm from resistive measurements (see Section S6 in Supplementary Information). Second, the strong confinement imposed by the 0.5 nm high and >10 µm long constriction (Figure 4f), which lies within the Odijk regime, leads to a very high free energy barrier preventing polymers from uncoiling and escaping along with the outward flow [36]. Lastly, computational simulations reveal that upon application of a voltage across the pore, a strong electric field near the microtrap edge is established, which further prevents the DNA from escaping through the nanometer sized gap (Figure S8 in Section S7 of Supplementary Information).

Equipped with a better understanding of this phenomenon, we can now revisit what occurs during the over‐pressurization of the microtrap. As shown in Figure 4a, the change in capture rate caused by preconcentration is a time‐dependent phenomenon. When the microtrap is first closed by applying pressure, the capture rate initially increases as the pressure forces the electrolyte out of the microtrap, while confining DNA molecules, thus increasing the molecular concentration within the microtrap. As the pressure within the microtrap equilibrates, the outward flow of electrolyte ceases, leading the capture rate to asymptotically approach a stable value.

Notably, when the microvalve pressure is lower than 5 psi, the capture rate exhibits a slight decrease following its initial increase, which can be attributed to two main factors. First, upon applying pressure to the microvalve, an outward flow is generated, subjecting the nanopore to cross‐flow. This cross‐flow has previously been shown to enhance the capture rate [30]. However, once the pressure within the microtrap equilibrates and the outward flow ceases, the capture rate correspondingly decreases [30]. Second, the outward flow causes DNA molecules to accumulate near the bottom of the microtrap, where the nanopore is located, creating a local concentration gradient. This temporarily increases the local concentration and enhances the capture rate. Over time, approximately two minutes, diffusion redistributes the DNA molecules throughout the microtrap, leading to a more uniform concentration and a slight reduction in the capture rate.

Additionally, at higher pressure (8 psi and 10 psi), the capture rate, calculated through the slope of cumulative number of events over time, is lower than the one measured at 5 psi valve pressure, an observation that may seem unexpected at first glance (see Figure 4a). However, monitoring the open pore ionic current reveals that at high valve pressures (>5 psi), excessive depletion of electrolyte from below the microtrap edges, increases the trap resistance, decreasing the voltage drop at the pore, thereby lowering the capture rate (see Figure 4b). Figure 4e reveals a ∼4X capture rate enhancement at 5 psi valve pressure after ∼2 min. Given that the mechanism of preconcentration here relies solely on reducing the sample volume by expelling electrolyte, a larger initial volume should, in theory, yield a higher preconcentration factor. To test this hypothesis, we conduct another test of applying high pressure on microtrap with 300 pL microtrap. In this case, a capture enhancement of ∼5X was observed when using a 300 pL microtrap at the same valve pressure (see Figure 4g). It is important to note that the thickness of the microvalve layer can also influence this enhancement factor, as the mechanism of the capture rate enhancement relies on the microtrap deformation. However, the thickness of the microtrap cannot be reduced below a certain threshold, as high valve pressure may cause PDMS rupture, leading to leakage between the microchannels.

### 2.6 Reducing TTR Using Preconcentration

Experiments presented in Figure 4 suggest that higher pressures can result in higher capture rates, which in turn suggest faster depletions and TTRs. This is an appealing feature given that other methods of reducing the TTR can introduce technical limitations such as ill‐resolved events due to limited temporal resolution at higher voltages, bad signal‐to‐noise ratio for increased pore size, or challenging device microfabrication and alignment for smaller microtrap volumes. We now explore when and how the preconcentration phenomenon described above (Figure 4) accelerates counting with microtraps.

**FIGURE 5:**
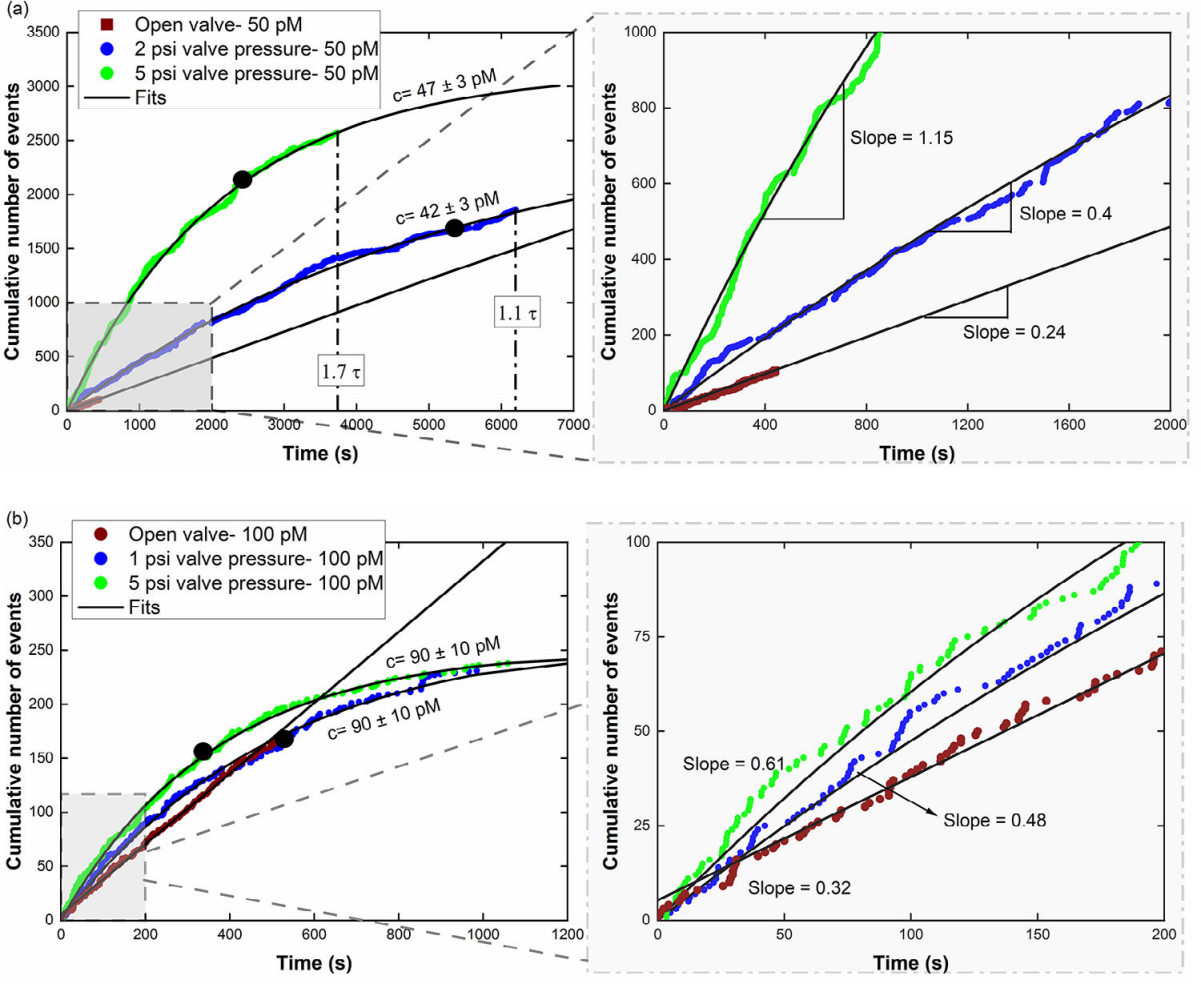
(a) Cumulative number of events over time measured using a 110 ± 7 pL microtrap volume for 50 pM of 2 kbp dsDNA samples at two different valve pressures. The nanopore's diameter and length are respectively 12.2 ± 0.5 nm and 12 ± 2 nm. The numbers of molecules within the microtrap at 2 psi and 5 psi valve pressure were predicted as 2850 ± 10, and 3100 ± 10 molecules, respectively. The TTR at 2 psi and 5 psi valve pressure was predicted respectively at 5370 ± 30 s and 2100 ± 10 s. (b) Cumulative number of events over time measured using a 5 ± 0.5 pL microtrap volume for 100 pM of 2 kbp dsDNA samples at 1 psi and 5 psi valve pressures. The nanopore's diameter and length are respectively 17.1 ± 0.5 nm and 12 ± 2 nm. The numbers of molecules within the microtrap at 1 psi and 5 psi valve pressure were measured as 260 ± 3 and 260 ± 1 molecules, respectively. The TTRs at 1 psi and 5 psi valve pressures were predicted to be 460 ± 10 s and 360 ± 3 s, respectively. The black circles are representative of the TTR, and the dash‐dot lines show the time for the end of the test. It should be noted that the green bands in panels (a) and (b) represent the expected numbers of molecule at the given concentrations, assuming a 10% sample preparation error. The R^2^ values were 0.99 for all fits. The c annotated on figures indicate the measured concentration.

As shown in Figure 5, and expected from the preconcentration mechanism discussed above, higher valve pressures reduced the TTR and enabled faster quantification. It must be noted that for this experiment, when the microvalve was pressurized to 5 psi, the bias voltage was applied 2 min after valve closure. This is mainly because, under 5 psi applied pressure, the capture rate changes for approximately one minute before equilibrating (see Section 2.5). If the bias voltage is applied from the very beginning, the time‐dependent increase in the capture rate alters the shape of the exponential curve, which can negatively affect the accuracy of curve fitting.

Figure 5a plots the cumulative 2 kbp dsDNA events captured from a 110 pL microtrap volume under pressures of 2 and 5 psi. The initial capture rates observed increase with higher applied pressures, highlighting the preconcentration phenomenon due to microtrap deformation and electrolyte expulsion. As such, the TTR (τ) measured by curve fitting analysis for 2 psi and 5 psi are 5370 ± 30 s and 2100 ± 10 s, respectively, showcasing pressure‐induced reduction in TTR. At 2 psi, the number of molecules inside the microtrap, N_0_, was measured to be 2850 ± 10 molecules, corresponding to a sample concentration of 42 ± 3 pM. At 5 psi valve pressure, the determined number of molecules was 3100 ± 10 molecules, equivalent to 47 ± 3 pM. Interestingly, both values agree well with the concentration of prepared sample (50 pM), even though their extraction were obtained from Equation 3, which assumes constant normalized capture rate (R) throughout the experiments. We note that the measured concentration at 5 psi provides a more reliable measurement than that at 2 psi because the experiment at 5 psi was conducted until 1.7 τ whereas it was conducted until 1.1 τ at 2 psi. A similar experiment was also performed using a smaller microtrap volume (5 ± 0.5 pL) with a 2 kbp dsDNA sample at 100 pM. Here, the microtrap was closed using either 1 psi or 5 psi valve pressures. The initial number of molecules, N_0_, at 1 psi and 5 psi was measured to be 260 ± 3 and 260 ± 1 molecules, respectively, corresponding to concentrations of 90 ± 10 pM and 90 ± 10 pM which agree well with the expected 2 kbp concentration. Interestingly, the TTR (𝞃) for 1 psi and 5 psi valve pressure measured to be respectively 460 ± 10 s and 360 ± 3 s, again highlighting an enhancement in the TTR due to preconcentration. However, the TTR enhancement is less significant, since the preconcentration factor is lessened at small volumes (see Section 2.5).

We lastly note that, despite the microtrap volume undergoing deformation during the preconcentration process, the calculation of the molecular concentration [c = N_0_/(N_A_V)] is related to the initial (uncompressed) microtrap volume. This is because the number of molecules within the microtrap remains constant after valve closure.

### 2.7 Protein Concentration Quantification

**FIGURE 6:**
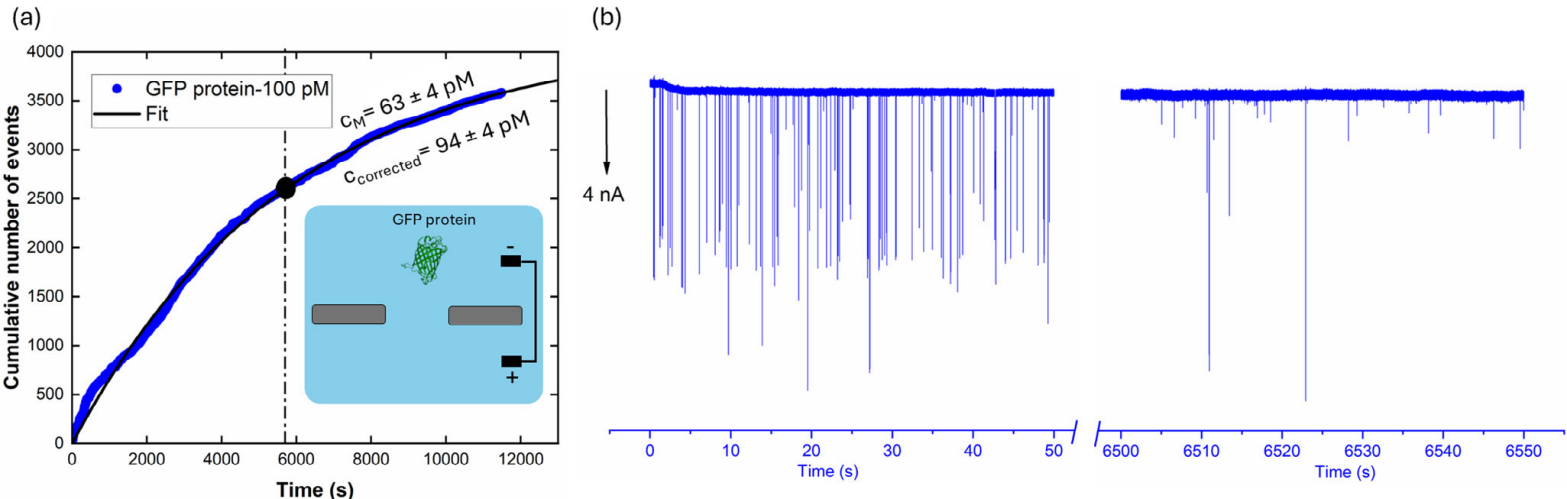
(a) Cumulative number of events over time measured using a 110 ± 7 pL microtrap volume for 100 pM of GFP protein samples when 2 psi valve pressure is applied. The inset demonstrates a schematic of a GFP translocation. The nanopore has a diameter of 7 nm and a nominal length of 12 nm. A bias voltage of 600 mV was applied, which was adjusted to 617 mV after valve closure. The number of molecules was measured to be 4150 ± 10, while the TTR was measured as 5790 ± 20 s. The R^2^ value of the fit was calculated as 0.99. The annotations c_M_ and c_corrected_ on the figure indicate the measured concentration and the concentration corrected for missed events, respectively. (b) Representative ionic current traces for the 100 pM GFP sample recorded at the beginning of the experiment and after 1.1τ, illustrating the depletion of microtrap over time.

While the majority of results presented until now focused on measuring the concentration of DNA samples, the microtrap nanopore sensor can also be used to quantify other biomolecules in solutions, including proteins. To demonstrate this capability, the platform was tested using green fluorescent protein (GFP), a 27 kDa protein with a beta‐barrel structure, which can be approximated as a cylinder measuring about 4.2 nm in length and 2.4 nm in diameter [37]. As shown in Figure 6a, when measuring with a microfluidic nanopore sensor with a closed valve, the cumulative number of GFP events follows the exponential decay model presented previously (Equation 1), highlighting the possibility of counting to depletion and inferring protein concentrations.

We determined, using our microfluidic nanopore system and similar analysis as before, the GFP concentration to be 63 ± 4 pM. This is close but somewhat off from the expected protein concentration, prepared to be 100 pM. This error is larger than the measurements for nucleic acid concentration, and the pipetting errors cannot explain this 40% discrepancy. Instead, we hypothesize that missed protein translocation events due to their fast passages and the finite temporal resolution of our nanopore system, can account for this disagreement. Indeed, employing a first‐passage time distribution for one‐dimensional diffusion like Plesa et al. [13]. can help infer the percentage of missed event and correct the measured concentration to be 94 ± 4 pM, which is in much better agreement with the expected GFP concentration (see Supplementary Information Section S3 for a detailed discussion). Another concern when measuring molecular concentration, particularly for proteins, is the adsorption of molecules onto PDMS surfaces, which can potentially lead to an underestimation of the measured concentration. However, our results demonstrate that the dominant source of concentration underestimation is the limited temporal resolution of the detection system rather than protein adsorption. A more detailed discussion is provided in Section S8 of the Supplementary Information.

Now that we established the concentration of GFP protein can accurately be measured if no events were missed, we discuss the kinetics of protein concentration compared to DNA. As expected, the TTR value measured for GFP, ∼2 hr is higher than for DNA, as electrophoretic mobility of GFP, and thus its normalized capture rate (R), is much lower than that of DNA. Consequently, any electrophoretically driven measurement will naturally yield substantially longer TTR values for proteins.

It must be noted that neither the incomplete resolution of molecular passage nor the low normalized capture rate observed in the protein concentration measurements is caused by the microtrap nanopore sensor itself. These effects are inherent to any ssNP measurement that relies on electrophoretic driving. The former issue has been addressed by ascertaining the percentage of missed events using FPT model which has previously been reported and used. To overcome the temporal resolution problem, strategies other than increasing the bandwidth has been suggested. For instance, it was shown that applying a pressure gradient across the nanopore in the appropriate direction can slow down the translocation of molecules, which improves the temporal resolution [21, 22, 38, 39, 40, 41]. In addition, creating an asymmetric salt concentration across the nanopore can both increase the capture rate and further slow the translocation time which partially mitigate both low temporal resolution and long TTR issues [17]. Another strategy is to modify ssNPs so that transient interactions with the protein increase its residence time within the pore which has been done recently by the Mayer's group [42]. In addition, biological nanopores coupled with motor proteins offer an effective means of controlling and slowing the translocation velocity of biomolecules [43]. As a complementary strategy, protein concentrations can also be quantified indirectly by converting target proteins into DNA proxies, whose abundance reflects the original protein level through immunoassay methods that previously demonstrated by our group and others [44, 45, 46].

## 3 Conclusion

We proposed an approach for quantifying molecular concentration using ssNPs that does not solely rely on establishing a stable nanopore capture rate, which can often be unstable and unpredictable [25]. This involves integrating the nanopore sensor within a microfluidic device capable of compartmentalizing a molecular sample in pL‐range volumes, enabling molecular counting to depletion. By utilizing this approach, the capture rate calibration is not required, thereby reducing systematic errors associated with quantification using calibrated capture rates. Importantly, the concentration quantification using nanopores becames independent of pore size measurements, which pose significant uncertainties due to either the overestimation of previously proposed models used for size calculations [47] or the assumption that pores are cylindrical [48]. In demonstrating this concept, the concentration of a nucleic acid fragment, 2 kbp dsDNA, and a protein, GFP, were measured by counting the finite number of molecules contained within a known microtrap volume. It should be noted that for protein concentration measurements, challenges such as low capture rate due to low electrophoretic mobility and the limited temporal resolution of the measurement system affected the time to response and accuracy, respectively. Moreover, these challenges would be overcome by using immunoassays to convert the target proteins into DNA proxies, which can then be detected with much higher speed and resolution [44]. Additionally, we examined the effects of the microtrap volume and operational parameters on the TTR, finding that smaller volumes and higher bias voltages, resulted in shorter response times. Furthermore, we demonstrated that leveraging the deformability of PDMS under high pressure enabled a real‐time increase in the concentration of the compartmentalized sample, speeding up the quantification process. We note that future optimization of the geometry and material of the proposed microtrap, can possibly further improve the TTR and lead to measuring samples at lower concentrations.

## 4 Materials and methods

### 4.1 Microfabrication and Device Assembly

The device integrates a silicon (Si) chip sandwiched between two PDMS multilayer components with embedded microchannels. The Si chips used in this study were purchased from Norcada Inc. and were employed without any further treatment in the fabrication of the test devices. For microtraps with 1–15 pL volumes, chips NXDB‐40B105V202 were utilized, comprising of a low stress silicon nitride (SiN) film deposited over a 200 µm thick 4 × 4 mm^2^ silicon frame, and featuring a 10 × 10 µm^2^ membrane with a nominal thickness of 20 ± 2 nm. For trap volumes higher than 100 pL, chips NXDB‐50B505A122 were used, equipped with an SiO_2_ noise reduction layer between the 50 × 50 µm^2^, 12±2 nm thick SiN membrane and the 5 × 5 mm^2^ support frame.

PDMS components were fabricated by soft lithography. Photomasks used to fabricate the master molds were designed in AutoCAD (Autodesk, Inc.). Following the fabrication of the master molds by standard photolithography using SU8‐2050 photoresist (Microchem Inc.) on polished silicon surfaces, each mold underwent profilometry measurements using Bruker DektakXT to determine the thickness of the features and microscopic imaging for confirmation of the dimensions using ImageJ software [49]. The molds were subsequently silanized with trichlorosilane (tridecafluoro‐1,1, 2, 2‐tetrahydrooctyl, Sigma Millipore) to facilitate PDMS removal. The valve and *trans* microchannels were prepared by casting PDMS (Sylgard 184 kit, Dow Corning) mixed at 10:1 (w:w) base to cross‐linker ratio in the molds at a thickness of 3 mm (see Figure S10 of Section S9 of Supplementary Information). The PDMS parts were degassed in a vacuum chamber for 40 min, then baked at 70°C for 3 h. To provide fluidic and electrical access, 750 µm holes were hand‐punched through PDMS using biopsy punches. Next, the *cis* microchannel was prepared by spin coating degassed PDMS on the silane‐treated silicon wafer master mold (10 s at 500 rpm followed by 30 s at 2000 rpm and 10 s at 0 rpm), followed by curing on a hot plate (80°C, 15 min). The spin‐coated *cis* microchannel layer was bonded to the PDMS ’valve microchannel’ using air plasma system (AutoGlow Plasma System, Glow Research) for 30 s at 48 W under 0.5 Torr pressure. The top dual‐layer component and the *trans* microchannel were each plasma bonded (30 s, 48 W) to a via layer, prepared by spin‐coating a thin layer of PDMS (40 µm thick) onto a blank silicon wafer, which was then hand‐punched using a 0.75 mm biopsy puncher to form the via (see Figure S10). The addition of a via layer serves to create a controlled distance that prevents bonding between the microtrap and the SiN layer on the chip. Another role of the via layer is the confinement of the electric field and electrolyte to a precise location on the SiN membrane, which has been shown to reduce the electrical noise of the nanopore [49] and helps to prevent soft breakdowns during nanopore fabrication by controlled breakdown (CBD) [50].

Following the preparation of three‐layer PDMS top component (valve, *cis* microchannel, and the via layer), it was plasma bonded (30 s, 48 W) to the chip to the membrane side. In order to precisely situate the PDMS composite atop the SiN membrane, all alignment steps were done using an OAI DUV/NUV mask aligner (Model 206). Subsequently, a thin layer of PDMS (200 ± 10 µm), gasket layer, was spin coated around the chip (10 s at 500 rpm followed by 30 s at 600 rpm) to compensate for its thickness and leave a leveled, smooth surface for bonding (see Figure S10). This thin layer was cured directly on a hot plate at 80°C for 20 min. Finally, the bottom PDMS component was plasma bonded to the etch pit side of the chip (30 s, 48 W), followed by a 2‐h oven bake at 80°C.

After completing the assembly, the entire device was subjected to plasma treatment (120 s, 42 W) to render the microchannels and chip surfaces hydrophilic. This step is crucial, given that the functionality of the device depends on maintaining electrical access even when the valve is actuated to lower the microtrap onto the SiN surface. Maintaining the proper level of PDMS hydrophilicity ensures that a nanometer‐scale film of electrolyte is maintained under the edge of the microtrap, which conserves an ionic current path (see Section S10 of Supplementary Information).

### 4.2 Nanopore Fabrication

The nanopores used in this study were fabricated in the SiN membrane within the microfluidic device using CBD, as previously reported [33, 34]. The device was mounted in a grounded metal box acting as a Faraday cage and was connected to solution‐containing vials via PEEK tubing. The first step was wetting the SiN membrane by sequentially loading the top and bottom microchannels with isopropyl alcohol (IPA, Sigma Millipore) and deionized (DI) water, using pressure‐driven flow. Then, a 1 M potassium chloride (KCl) solution (BP366 – Fisher Scientific), buffered with 10 mM 4‐(2‐hydroxyethyl)‐1‐piperazineethanesulfonic acid (HEPES), (BP310 – Fisher Scientific), with a conductivity of 11.2 S/m and a pH 8, was loaded into the microchannel. Next, the SiN membrane was exposed to high electric fields via a ramped transmembrane bias voltage applied by a custom build circuit (3 V/min up to maximum 5 V followed by 0.75 V/min to maximum 18 V), through a pair of Ag/AgCl electrodes (Cat number, 041456.G1, Thermo scientific), inserted into the microchannels through their corresponding punched holes, one within the top microchannel and the other within the bottom microchannel. Upon the formation of the nanopore, a sudden jump in measured leakage current was observed triggering the software to turn off the bias voltage. This process typically results in the creation of very small nanopores (<2 nm) that subsequently require enlarging to the desired size (5–20 nm range) [51, 52], which involves applying moderate voltage pulses with the amplitude of 2–8 V and duration of 1 s, while the microchannel was filled with 3.6 M lithium chloride (LiCl, L121 – Fisher Scientific), buffered with 10 mM HEPES pH 8 and conductivity of 16.8 S/m. All results presented in this study were obtained using 15 independently CBD‐fabricated nanopores to ensure the consistency of results between many different devices and nanopores, with their corresponding dimensions reported in the manuscript. An example of current and voltage data during nanopore fabrication, enlargement, and size measurement (I–V curve) for a nanopore in this study is provided in Section S11 of Supplementary Information.

### 4.3 Sample Preparation and Introduction

Double‐stranded DNA and GFP samples at desired concentrations were prepared by diluting molecular sample in a 3.6 M LiCl solution. The resulting mixture was homogenized by gentle pipette mixing to ensure uniform distribution of the molecular sample. Although the dsDNA (NoLimits, Thermo Scientific) and GFP sample stocks were stored in 1.5 mL microcentrifuge tubes at 4°C, they were allowed to equilibrate to room temperature before any experiment. All experiments were conducted at room temperature. The prepared sample was then introduced into the *cis* microchannel of the microfluidic device, while the bottom *trans* microchannel was kept filled with 3.6 M LiCl solution under no flow. A negative bias voltage was then applied to the *cis* microchannel using the Ag/AgCl electrodes inserted in the *cis* and *trans* microchannels and connected to the headstage of a single‐channel patch clamp amplifier (Axopatch 200B, Molecular Devices) while the time‐series electrical current recordings were acquired.

After actuation of the on‐chip valve using a pressure regulator to close the trap, the conductance of the system decreases due to the addition of a resistance in series between the electrodes (see Figure 1a,b). The reduction in measured ionic current for a given voltage leads to a reduced electric field strength through the nanopore, which in turn effectively lowers the nanopore capture rate compared to when the trap is open. To operate the pore under the same electrical conditions when the microtrap is open or closed, the bias voltage was adjusted after microtrap closure to re‐establish the same ionic current open baseline, which ensures that the electric field strength within the nanopore is at a level similar to when the microtrap is open.

### 4.4 Data Acquisition and Analysis

Once the DNA solution was loaded in the microfluidic device and the bias voltage was applied, the ionic current was sampled at 250 kHz, by interfacing the current amplifier with a custom‐written LabVIEW‐based software and a DAQ card (National Instrument USB‐6353). Ionic current data were low pass filtered at 100 kHz using a 4‐pole Bessel filter to reduce parasitic high frequency noise.

A custom‐written code based on a CUSUM algorithm was employed to perform analysis on the acquired ionic current data, wherein every abrupt deviation from baseline was indexed and considered as a potential DNA translocation event [53, 54]. The indexed events were filtered based on their translocation time and maximum blockage, to remove any ambiguous events. Afterward, the capture rate of molecules could be calculated from the filtered data, by fitting the slope of the cumulative number of events as a function of time.

## Conflicts of Interest

The authors declare no conflicts of interest.

## Supporting information




**Supporting File**: smtd70924‐sup‐0001‐SuppMat.docx.

## Data Availability

The data that support the findings of this study are available from the corresponding author upon reasonable request.
