# Supplementary material for: Microfluidic Sample Compartmentalization for Biomolecular Concentration Quantification Using a Nanopore Sensor
Source: Small Methods. 2026 Aug 11;10(18):e70924. doi: 10.1002/smtd.70924 (PMC13599688; doi:10.1002/smtd.70924)
Supplement: Supplementary file 1 [file SMTD-10-e70924-s001.docx]

Supplementary information for:

**Microfluidic sample compartmentalization for biomolecular concentration quantification using a nanopore sensor**

Morteza Safari^2^, Juliana Chawich^1^, Ali Najafi Sohi^1^, Martin Charron^1^, Vincent Tabard-Cossa^1, 2^, Michel Godin^1, 2^

^1^ Department of Physics, University of Ottawa, 150 Louis-Pasteur, Ottawa, Ontario, Canada K1N 6N5
^2^ Ottawa-Carleton Institute for Biomedical Engineering, Ottawa, Ontario, Canada

**Table of Contents**

[S1. Capture rate-based concentration measurement	2](#_Toc233364848)

[S2. Calculation of Error propagation	2](#_Toc233364849)

[S3. Calculation of percentage of missed events using First Passage time model	3](#_Toc233364850)

[S4. Minimum duration of the quantification experiment	5](#_Toc233364851)

[S4.1 Oscillating normalized capture rate	7](#_Toc233364852)

[S4.2 Drifting normalized capture rate	8](#_Toc233364853)

[S4.3 Randomly varying normalized capture rate	10](#_Toc233364854)

[S5. Brownian dynamics simulations for TTR estimation	12](#_Toc233364855)

[S6. Valve characterization	13](#_Toc233364856)

[S7. Effect of microtrap on electric field	14](#_Toc233364857)

[S8. Adsorption of protein to the surface of PDMS	17](#_Toc233364858)

[S9. Device assembly	20](#_Toc233364859)

[S10. Role of hydrophilicity in device function	20](#_Toc233364860)

[S11. Nanopore fabrication, enlargement, and size measurement data	22](#_Toc233364861)

## S1. Capture rate-based concentration measurement

*Figure S1. (a) Cumulative single molecule counts over time for 2 kbp dsDNA samples of varying concentrations measured on a single nanopore at 400 mV bias voltage. (b) Measured concentrations determined using the conventional capture rate method at different time points during the experiment. Pore 1 has a diameter of 11 ± 0.3 nm and a length of 12 nm, while Pore 2 has a diameter of 9.5 ± 0.4 nm and a length of 20 ± 2 nm*

## S2. Calculation of Error propagation

Uncertainty for measured concentration was calculated based on uncertainty in curve fitting and determination of the microtrap volume. The former is mainly influenced by the variability in the normalized capture rate during the experiment and is represented by the standard error of the curve fitting. The latter, however, arises from uncertainties in the device fabrication process and the deformation of the microtrap under pressure. The uncertainty in the volume was calculated based on uncertainties in determination of height and diameter of the microtrap. The height uncertainty was estimated from the variation in thickness measured across the wafer. For the diameter of the microtrap, the uncertainty was estimated from the variation in measured diameters across the wafer using microscopy (ImageJ). Then, considering the microtrap as a cylinder, so $V=\pi r^{2}L$, the uncertainty in the volume was calculated through error propagation;

$$\frac{\mathrm{dV}}{V}=\sqrt{\left( \frac{\partial V}{\partial r}\mathrm{dr} \right)^{2}+\left( \frac{\partial V}{\partial L}\mathrm{dL} \right)^{2}} , (Eq.S1)$$

when V, r, and L are volume, radius, and length of the microtrap respectively, and their corresponding uncertainties are denoted as dV, dr, and dL. Moreover, N_0_ and dN_0_ represent the initial number of molecules and the uncertainty associated with it, respectively. Finally, the uncertainty in concentration of molecules, which is $c=\frac{N_{0}}{N_{A}V}$, was obtained by

$$\frac{\mathrm{dc}}{c}=\sqrt{\left( \frac{\mathrm{dN}_{0}}{N_{0}} \right)^{2}+\left( \frac{\mathrm{dV}}{V} \right)^{2}} \left( Eq.S2 \right)$$

## S3. Calculation of percentage of missed events using First Passage time model

To ensure that no molecule is being lost due to limited temporal resolution of the system, we use First Passage time, FPT, model to calculate the fraction of missed events described in the paper by Talaga and Li, which infers the percentage of missed events using the FPT for one-dimensional diffusion^1^. In this venue, the probability density function of 1D first passage time model is

$$\mathrm{PDF}_{\mathrm{FPTD}}\left( \tau\right)=\frac{h_{\mathrm{eff}}}{\left( 4\pi D\tau^{3} \right)^{\frac{1}{2}}}e^{-\frac{\left( h_{\mathrm{eff}}-v\tau\right)^{2}}{4D\tau}} \left( Eq.S3 \right)$$

when h_eff_, D, $\nu$, and 𝞃 are effective sensing length which here assumed to be equal to nanopore length, diffusion coefficient, drift velocity, and the dwell time of the molecule, respectively. By fitting this model on experimental dwell time data, one can measure the diffusion coefficient and translocation velocity, assuming the effective sensing length is known. In this study, the effective sensing length, h_eff_, is approximated differently depending on the relative size of the molecule and the nanopore. For molecules shorter than the pore, such as proteins, is approximately equal to the pore length. For molecules longer than the pore, h_eff_, is taken as the sum of the molecular length and the pore length. Using this method, we determined the fraction of lost events in the experiments, particularly under conditions with larger nanopores and higher applied voltages, where unresolved molecules and missed events are more likely due to the limited bandwidth of the measurement system. In this context, for the 25 pM experiment performed at 872 mV with a 9.2 nm pore (blue graph in Figure 2b) and the 35 pM experiment conducted at 634 mV with a 25 nm pore (green graph in Figure 2b), the dwell time distributions were analyzed, and the fraction of events occurring before 10 µs was determined using the FPT model. For these calculations, the effective sensing length was taken as h_eff_ = 692 nm, corresponding to the sum of the 2 kbp DNA length and the nominal pore length as suggested by Li et al^2^. As shown in Figure S2 in both case the fraction of lost events is negligible meaning that the measured concentration is not significantly affected by the limited temporal resolution. We note that the 10 µs threshold was not selected solely based on the temporal resolution of the amplifier; rather, it was chosen based on the minimum detectable pulse duration, which represents the shortest translocation event that can be fully resolved.

*Figure S2. Dwell time distributions for 2 kbp dsDNA translocation under various experimental conditions, along with the fitted FPT model. (a) 25 pM experiment performed at 872 mV with a 9.2 nm pore (blue curve, corresponding to Figure 2b) and 35 pM experiment conducted at 634 mV with a 25 nm pore (green curve, corresponding to Figure 2b). (c) Dwell time distribution measured using a 110 ± 7 pL microtrap volume for 100 pM of GFP protein samples when 2 psi valve pressure is applied along with the fitted FTP model (corresponding to data presented in Figure 6). The inset in (c) demonstrate an example of fully resolved GFP events. (d) Calculating of the h_eff_ for both DNA and protein experiments. It should be noted that the two diagrams in panel (d) are plotted on different scales.*

Losing events due to limited temporal resolution becomes an even more significant challenge when testing with proteins. In our experiments with GFP, we observed a biased dwell-time distribution, suggesting that a substantial fraction of translocations are lost due to the temporal resolution. To quantify these missed events, we applied the FPT model, with the modification that the effective sensing length was taken as 12 nm, corresponding to the nominal thickness of the membrane used for nanopore fabrication. Prior to curve fitting it must be noted the dataset used must only contain fully resolved protein translocation events, which include events with $\tau$ > 50 µs (Figure 6c). In this vein, as shown by Plesa et al. unresolved events must not be included in the curve fitting as the real dwell time ascertain from these events can different then the one calculated through nanopore data analysis^3^. Moreover, in our experiments we observed many protein translocation events that were significantly longer than the typical GFP translocation times. These prolonged events are likely caused by nanopore–protein interactions and are not captured by the FPT model. Therefore, to reduce fitting uncertainties, all translocation events longer than 1000 µs were removed, and only fully resolved events were included in the analysis. By fitting the experimental dwell time distribution on FTP model, the diffusion coefficient and drift velocity were ascertained respectively as 0.7 ± 0.003 nm^2^/µs and 0.05 ± 0.0003 nm/µs. The diffusion coefficient of GFP within a nanopore determined in this study agrees with the value reported by Waduge et al. but in both cases, it is 100–150 times smaller than the bulk diffusion coefficient^4^. The primary reason for the discrepancy between the in-pore diffusion coefficient and bulk values is the confinement within the nanopore, combined with protein–nanopore interactions, which collectively reduce the effective diffusion coefficient, consistent with previous reports^4–6^.Using these parameters, the fraction of events with dwell times less than 50 µs, corresponding to the cutoff below which events were detected in an unresolved manner, can be calculated as 33 %, assuming the effective pore length is 12 nm (see Figure S2c). It should be noted that the temporal resolution cutoff for proteins is higher than that for DNA. This is primarily due to the smaller size of proteins, which leads to an increase in the minimum detectable pulse duration. As previously reported, smaller molecules exhibit longer minimum detectable pulse durations, reflecting the greater difficulty in fully resolving their translocation events^3^.

## S4. Minimum duration of the quantification experiment

As highlighted in the main paper, the reliability of quantification results using curve fitting depends on the duration of the experiment, which is shown to be a function of the time-to-response, TTR. Our experimental results indicate that this parameter is heavily influenced by the change in the normalized capture rate during the experiment which can be characterize by R^2^ of the fit. Figure S3 depicts the curve fitting error versus number of datapoints under different testing conditions and the R^2^ values. To calculate the error at different data points, the last data point of the full dataset was sequentially removed. After each removal, the curve was refitted, and the process was repeated until the exponential equation (Eq. 1) could no longer be accurately fitted to the reduced dataset. This approach allowed us to evaluate the sensitivity of the fitting accuracy to the amount of available data and to determine the minimum number of data points required for reliable curve fitting. In this venue, tests with minimal normalized capture rate variability, characterized by higher R^2^ values, the minimum time for experiment is respectively approximately 1.2 τ, where the fitting error is below 10%. These time scales are equivalent of counting approximately 75 % of initial number of molecules. It is important to note that, while the curve fitting for these tests achieved R^2^ = 0.99, there was still some variability in the normalized capture rate as can be seen in Figures 2b, 5a, and 5b. However, these variabilities were not significant enough to disrupt the curve fitting process. Therefore, microtrap-based quantification can tolerate some variability in normalized capture rate.

Surprisingly, for 250 pM and 500 pM tests demonstrated in Figure 2d, blue and green graphs in Figure S3, the minimum runtime significantly increased to 1.7 τ and 2.2 τ, respectively. This is mainly because in both of these tests the normalized capture rate tends to exhibit random oscillation, characterize by lower R^2^ values. Therefore, an accurate curve fitting requires running the experiment for longer time, which correspond to counting 80 and 85% of initial number of molecules, respectively. Therefore, it can be concluded that even with variable normalized capture rate the concentration can be determined with the cost being running longer quantification experiment.

*Figure S3. The normalized error of the curve fitting at different time of the experiment (a) and different fraction of captured molecules (b) for four different testing condition. The green area for both graph shows where the normalized error of fitting is below 5%.*

While analyzing experimental data provides valuable insights into TTR, performing a systematic study on effects of normalized capture rate variation on TTR is challenging. In this vein, the time-varying normalized capture rate was simulated using non-homogeneous Poisson processes. Three time-dependent models were considered: oscillatory, drifting, and randomly variable normalized capture rates, R. These models were substituted into,

$J=Rc\left( t \right)=\frac{\mathrm{RN}\left( t \right)}{V} , (Eq.S4)$

with J, c, and $V$ respectively being the capture rate, concentration, and the microtrap volume, replacing the assumption of a constant normalized capture rate. After solving the resulting equation and using the obtained expression for N_cap_, simulated event data were generated, and Eq. 1 was fitted to the simulated results. Finally, the error in concentration measurement was quantified by comparing the initial number of molecules with the predicted initial count obtained through curve fitting.

### S4.1 Oscillating normalized capture rate

First, oscillating normalized capture rate variation was considered, which was common in the experimental data presented in this paper (see Figures 2b, 5a and 5b). Such oscillations were modeled by considering $R\left( t \right)=R_{0}\left( 1+A\sin(\omega t) \right)$ with $A\leq1$ and $\omega$ are respectively being amplitude and frequency of the oscillation. In this case, substituting R(t) in Eq S4 gives,

$$N_{\mathrm{cap}}\left( t \right)=N_{0}\left( 1-\exp\left( -\frac{R_{0}}{V}(t+\frac{A}{\omega}(1-cos(\omega t)) \right) \right) (Eq. S5)$$

See example plotted here: <https://www.desmos.com/calculator/qyllp79xjc>

To examine the effect of oscillation on curve fitting results, Eq. 1 was fitted on Eq. S5 at different data point, amplitude and frequency. Then, the normalized error of curve fitting was calculated along with the R^2^ of the fitted curve. Figure S4a show the cumulative number of simulated events at different oscillation frequencies when A=1. The results indicate that the worst-case scenario for curve fitting occurs when the amplitude is highest, which is A=1, and the frequency is lowest. Figure S4b further confirms this intuition as by demonstrating the normalized error in curve fitting at different amplitude and normalized frequency of oscillation, τω, when N_0_=1000. The normalized frequency of oscillation, τω, represents the ratio of the oscillation period to τ, which characterizes the system's response time, TTR. As can be seen, when τω>10, by increasing τω, the influence of oscillations on the curve fitting results diminishes. This is reasonable because at higher τω, the oscillation period is much shorter than the TTR, so the system has sufficient time to adjust, leading to minimal impact on the fitting results.

However, when 1<τω<10, the oscillation period is comparable to TTR, τ, leading to mixed impact on normalized error. Starting from τω=1 to a threshold, the normalized error increase as τω increases. This might seem counterintuitive, but when τω=1 both oscillation period and the TTR are in a same scale meaning that the oscillation just starts to interfere with the exponential curve. Hence, increasing τω, makes the oscillation shorter compared to the response time, causing more interference with fitting. However, after reaching to a threshold, which is around τω=2 in left panel of Figure S4b, the normalized error starts to decrease and reaches to zero at a critical τω or (τω)_C_, which is around 4 in the left panel of Figure S4b. This happens when the oscillation period and TTR reach a balance where the variations cancel out over the integration process, allowing the fitting equation to perfectly approximate the data. After this threshold, the normalized error increases as the oscillations start to significantly distort the cumulative event count again, deviating further from the purely exponential model used for fitting. In this situation, the system no longer averages out the oscillations effectively, and their impact becomes more pronounced in the data, thereby increasing the normalized error. It must be noted that, by running the simulation for longer time, not only the normalized error significantly decreases, but also the (τω)_C_ also decrease (see Figure S4b). It must be noted that τω cannot be lower than 1. If it is, the oscillation frequency falls outside the TTR, rendering the oscillation irrelevant for curve fitting. Hence, the oscillation does not significantly impact the curve fitting results.

*Figure S4. (a) Cumulative number of simulated events at different normalized frequency of oscillation conditions when A=1. (b) The normalized error of curve fitting for various oscillation conditions after counting for time scales.*

### S4.2 Drifting normalized capture rate

Next, we assume that the normalized capture rate drift during experiment according to $R\left( t \right)=r_{o}+rt$. In this case, the number of captured molecules is given by,

$$N_{\mathrm{cap}}\left( t \right)=N_{o}(1-exp(- \frac{R_{o}t+\frac{1}{2}rt^{2}}{V})). (Eq. S6)$$

Similar to the previous section, the normalized error was calculated at different simulation times, and drift conditions. In all of our simulations, the R_0_=1 Hz nM^-1^, which is is a typical value for solid-state nanopores. As shown in Figure S5b, when the drifting rate, r, is significantly smaller than the R_0_, it has a minor impact on curve fitting. This is mainly because in this case the second term the exponent, $\frac{1}{2}rt^{2}$, becomes very small making the drift negligible. However, a small increase in the drift rate causes the normalized error to rise significantly (see Figures S5b-S5d). This is because the second term in the exponent is second order with respect to t, meaning it increases faster than the first term, $R_{o}t$, as time progresses. Beyond a certain threshold, which depend on the time of the simulation, the normalized error begins to decrease as the drift rate increases, which might seem counterintuitive since in this regime, the exponential behavior of N_cap_(t) is primary dictated by the $\frac{1}{2}rt^{2}$term. However, despite this shift, the overall curve remains similar to Eq. 1, with an approximately similar N_0_ but a different time constant resulting in a better fit to the data, reducing the normalized error (see Figures S5b-S5d). For example, as shown in Figure S5c, with τ = 1000 s and N_0_ = 1000 molecules, we would expect that without drift, 63% or 630 molecules should be counted in the first 1000 s, which is the case in Figure S5a where the drift is negligible. However, Figure S5c indicates that the drifting data asymptote to approximately 1000 molecules after ~ 200 s, with the τ of the fitted curve being ~ 170 s. Therefore, at higher drift rate, the curve, is equivalent to the Eq .3 but with shorter TTR.

*Figure S5. Cumulative number of simulated events at different drifting conditions at R0=1 Hz nM^-1^ and (a) r = 0.0001 Hz nM^-1^s^-1^, (b) r = 0.01 Hz nM^-1^s^-1^, and (c) r = 0.1 Hz nM^-1^s^-1^. (d) The normalized error of curve fitting for various drifting condition and simulation time. In all simulations the R_0_ = 1 Hz nM^-1^, N0 = 1000 events, and τ = V/R_0_ = 1000 s.*

### S4.3 Randomly varying normalized capture rate

Lastly, the effect of random variation in normalized capture rate on curve fitting was explored. For this purpose, it was assumed that the normalized capture rate, R(t), varies according to a biased random walk. In this case, $R\left( t+dt \right)=R\left( t \right)+\sigma\sqrt{\mathrm{dt}} \vec{\boldsymbol{\omega}}$, with $\sigma$ and $\vec{\boldsymbol{\omega}}$ are respectively noise intensity and a random vector sampled from a normal distribution with zero mean and a standard deviation of 1. Substituting this in Eq. S4 yields

$$N_{\mathrm{cap}}=N_{0}\left( 1-\exp\left( \int_{0}^{t} R\left( t \right)\mathrm{dt} \right) \right). (Eq. S7)$$

After generating a random walk for R(t) when dt = 0.1 s, the integral in Eq. S7 was calculated numerically and cumulative number of simulated events were obtained. Figure S6 demonstrate the normalized error at different noise intensity and time and each graph contain the normalized error for 10 simulated random walks. As expected, noise intensity significantly impacts the curve-fitting results, but its effect diminishes when the simulation is run for a longer duration. It is important to note that, in practice, noise intensity rarely exceeds 0.01, and the duration of variation is typically shorter. As a result, a truly random walk variation in the normalized capture rate is rarely observed in both experiments performed in this study and the literature^7^.

Figure S6. (a) The variation of normalized capture rate over time at different noise intensity. Cumulative number of simulated events at (b) $\sigma=$ 0.001 and (c) $\sigma=$ 0.01. The normalized error of curve fitting after counting for (d) τ seconds, (e) 2τ sec, and (f) 5τ sec. In all simulations the R_0_ = 1 Hz nM^-1^, N_0_ = 1000 events, and τ = 1000 sec.

After evaluating three time-varying models for the normalized capture rate, it was observed that in most cases, curve fitting results exhibit an error greater than 20% if counting is limited to 2τ. It is important to note that the time-varying models used here are exaggerated representations of experimental conditions. In practice, as demonstrated in the main paper, the minimum required counting time is likely shorter than 2τ, and the measurement error is lower than what was simulated.

## S5. Brownian dynamics simulations for TTR estimation

It is essential to model kinetics of molecular capture within the microtrap to estimate the TTR. Given that under all experimental conditions in this study, the molecular capture process is governed solely by a diffusion-limited regime^7^, the motion of the molecules can be described using a Brownian motion model and electrophoretic drift. We observed that at high salt concentrations, dsDNA molecules tend to exist as a blob, so it is reasonable to model them as blobs. However, this assumption does not hold within the capture volume of the nanopore, where the molecule experiences significant elongation and non-equilibrium deformation due to strong electrophoretic forces. Hence, our model focuses on simulating the motion of spherical nanoparticles within a cylindrical domain, representing a microtrap, and specifically outside the capture volume, where particles are influenced solely by Brownian motion. To this end, particles were initially randomly distributed within this domain, and then the position of each particle was updated at each time step based on the translational diffusion equation,

$${\vec{\mathbf{r}}}_{t+\Delta t}{=\vec{\mathbf{r}}}_{t}+\sqrt{2D\Delta t} \vec{\boldsymbol{\omega}}\mathbf{,} \mathbf{(}Eq.S8\mathbf{)}$$

where $\vec{\mathbf{r}}$ = ($\mathbf{x}\hat{\mathbf{x}}$, $\mathbf{y}\hat{\mathbf{y}}$, $\mathbf{z}\hat{\mathbf{z}}$) is the location of particle in Cartesian coordination, $\hat{\mathbf{x}}$, $\hat{\mathbf{y}}$, and $\hat{\mathbf{z}}$ are unit vectors of the coordinate system, D, $\Delta t$, and $\vec{\boldsymbol{\omega}}$ are respectively the diffusion coefficients, the time step, and a random vector sampled from a normal distribution with zero mean and a standard deviation of 1. It must be noted that in the simulations, DNA molecules were modeled as point particles whose transport was governed by their diffusion coefficient. Consequently, polymer-specific properties such as blob size, persistence length, and radius of gyration were not explicitly incorporated into the model.

Eq. S8 was solved numerically using Python in the Jupyter Notebook environment for different concentrations of the particles with a time step $\Delta t={10}^{-7}s$. The time step was chosen to ensure that the maximum displacement of the molecules per step is smaller than the nanopore capture radius. This approach ensures that molecules are accurately captured as they enter the capture volume, preventing them from bypassing it during the simulation. The maximum displacement in the simulation calculated using, $\left| \Delta\vec{\mathbf{r}} \right|=\left| \vec{v}_{m} \right|\Delta t$ when $\left| \vec{v}_{m} \right|$ is the magnitude of maximum browning motion velocity which is $\left| \vec{v}_{m} \right|=\left( 2D \right)^{0.5}$. In this study, D considered as $5 \mu m^{2} s^{-1}$ for 2kbp dsDNA^8^. To further refine the simulation, boundary conditions were implemented to ensure accurate particle behavior within the system. For walls, reflective boundary conditions were applied to ensure that particles remained within the cylindrical volume. At the base of the cylinder, a semi-spherical absorbing boundary condition was chosen, to represent the capture volume of the nanopore. Once a particle diffuses to this semi-spherical surface, it is idealized as captured and is subsequently removed from the simulation domain. This simplification allows us to model the initial diffusion process, acknowledging that the actual capture event may involve additional complexities such as electrophoretic forces and particle deformation. It must be noted that the calculation of the capture radius of the nanopore is necessary to solve Eq. S8 with correct boundary condition. To this end, the capture volume is defined as a region where the electrophoretic force resulted from strong voltage drop near the nanopore overcome the Brownian motion velocities of molecules and calculated using^8^

$$r^{*}=\frac{\mu_{e}\Delta\phi d^{2}}{8LD} (Eq.S9)$$

Where $\phi$, d, and L are respectively electric potential, pore diameter, and length. Also, $\mu_{e}$ = ${10}^{3}\mu m^{2} V^{-1}s^{-1}$ is the electrophoretic mobility of DNA and independent of the length of the DNA^8^.

## S6. Valve characterization

The microvalve that contains the microtrap is essential for the functionality of the device presented in this study. Upon valve closure, the gap between the microtrap and the microchannel bottom must be small enough to prevent molecules from escaping or entering, yet large enough to maintain electrical access to the nanopore. This gap distance can be tuned via two parameters, including the microvalve pressure and the level of surface hydrophilicity, the effects of which was examined by performing fluorescence microscopy studies and electrical resistance measurements.

The minimum pressure for valve closure was determined using fluorescence microscopy. For this purpose, the microtrap was filled with fluorescein, and the valve was pressurized to 5 psi or higher, ensuring that the microtrap was fully closed at these pressures. Afterwards, the remaining fluorescein was washed from the microchannel using DI water. A small flow of DI water was applied at the inlet while gradually reducing the valve pressure. The pressure just before the microtrap contents were washed away by the DI water flow is considered to be the minimum valve closure pressure. As can be seen in Figure S6, above 1 psi the microtrap still allowed molecules escaping given that the intensity of fluorescence did not decrease.

Figure S7. (a) Fluorescent images of the microtrap filled with fluorescein in the presence of a flow of DI water at different microvalve pressures to determine the valve closure pressure The scale bar indicates 40 μm. (b) Effect of the microvalve pressure on the nanopore baseline. The experiments were performed with a nanopore with size and length of respectively 13.6 ± 0.3 nm and 20 ± 2 nm at 200 mV bias voltage.

To prevent molecules escaping from the microtrap, the gap between the microchannel bottom and the microtrap edge must be smaller than the radius of gyration of dsDNA. Since this gap was unmeasurable by microscopy, it was determined by analyzing the system's electrical resistance in both open and closed valve conditions, with the nanopore present. In this vein, the resistance between two cylinders with radius of $r_{\mathrm{out}}$ and $r_{\mathrm{in}}$ across which a voltage is applied can be calculated through

$$R=\frac{1}{2\pi\sigma L}\ln(\frac{r_{o}}{r_{i}}) (Eq.S10)$$

where R and L are respectively representing the electrical resistance and the gap between the microtrap and the microchannel. Using Eq. S10 and the resistance from Figure S7b, the electrolyte layer thickness beneath the microtrap was calculated to be less than 1 nm, thus preventing the DNA blobs from escaping.

## S7. Effect of microtrap on electric field

Upon closing the valve, the nanometer-sized gap (<1 nm) between the microtrap edge and the microchannel generated a high electric field. In this section, we assess the strength of this field and investigate the potential expansion of the capture radius due to this effect. Hence, two dimensional (2D) symmetric finite element simulations were performed in COMSOL Multiphysics V6. In this framework, two reservoirs were connected through a nanopore with the diameter and length of 15 nm and 20 nm, respectively. The electric field was calculated through numerically solving the Poisson equation, $\nabla^{2}\phi=0$, where φ is electrostatic potential. Then, the electric field E ascertained by solving $E=-\nabla\phi$ in COMSOL Multiphysics^9^. Since all the experiments were performed in high salt concentrations, the concentration of cations and anions is equal in the bulk, and the mobile charge density is negligible. We also noted that in reality the size of the top and bottom reservoir is different, but since that is not affecting the electric field of the nanopore, the two reservoirs were modeled with the same size.

The results of the computational simulations revealed that the gap focuses the electric field, creating a repulsion zone where the electrophoretic force dominates over diffusion, preventing molecules from escaping (Figure S8). However, the repulsion zone cannot overlap with the nanopore’s capture volume, as it is located at least 5–10 µm away from the nanopore. This distance cannot be experimentally determined due to the stochastic nature of the CBD method, which leaves the nanopore’s exact location on the membrane unknown. The simulation results indicate that the electric field in the repulsion zone is approximately 10% of that around the nanopore when the gap between the microtrap and microchannel is 0.5 nm. This is extending the repulsion zone by about >10 nm within the microtrap, making any overlap with the capture volume impossible (see Figure S8). Yet, such high electric region still generates strong electrophoretic force that prevents molecule escaping the microtrap.

*Figure S8. Amplitude of the electric field at the green line level and at different electrolyte layer thickness beneath the microtrap, calculated using numerical simulation with an applied bias voltage of 400 mV. The diameter and length of the nanopore for this simulation were respectively 15 and 20 nm. It must be noted that the top schematic is not drawn to scale.*

## S8. Adsorption of protein to the surface of PDMS

While in the main text we claim that the main source of error in underestimation of protein concentration is temporal resolution of or amplifier, there might be other possible source of missing molecules. One concern is adsorption of molecules to PDMS surface which might cause underestimating the concentration. To shed light on this, this section is aiming to proof the main source of missing events when doing protein measurement is temporal resolution and while the adsorption might be a small factor, its effect if negligible. We noted that quantifying the exact percentage of proteins adsorbed onto the PDMS wall is challenging for two main reasons. First, deconvoluting protein adsorption from missed fast events is difficult to examine when using microtrap-based concentration quantification. Second, fluorescence microscopy on fluorescent protein does not provide a directly comparable measurement in our case, because we work at very low protein concentrations, where adsorption of only a small number of proteins can lead to large variations in the estimated concentration, while it cannot be measured using fluorescent microscopy.

To this end, rather than quantifying the absolute percentage of proteins adsorbed onto PDMS, we focused on demonstrating that the dominant contribution to missed events arises from the limited temporal resolution of the measurement. To this end, we performed two control experiments in which protein samples were loaded into a chemically inert fluidic cell, and the percentage of missed events was measured under the same detection conditions, same applied bias voltage and nanopore size and conductance. Under these conditions, the majority of missed events are expected to originate from the limited temporal resolution of the nanopore measurement as the fluidic cell is chemically inert. Moreover, if there was sticking molecules to fluidic cell, that does not affect the percentage of missed events due to the temporal resolution since that is only depends on the dwell time distribution of molecules.

Interestingly, the percentage of missed events measured in the passivated fluidic cell with a nanopore with approximately similar size (8 nm in diameter and 12 nm nominal length) at the same nanopore conductance was very similar to that observed for measurements performed using nanopores of in PDMS microfluidic device. As can be seen, the percentage of missed events in the flow cell control experiment calculated as 36% which is agree well with the result obtained in PDMS microfluidic device, which was 33% (see Figure S9a). Although a small difference was observed between the measurements, this discrepancy can arise from several factors, including pore-to-pore variability resulting from the use of different nanopores. This agreement strongly suggests that fast translocation events falling below the detection bandwidth constitute the dominant source of missed events. Consequently, while some residual protein adsorption cannot be completely excluded, its contribution is expected to be small compared with losses arising from limited temporal resolution. Interestingly, we have shown that the percentage of missed events can be lowered by reducing the applied bias voltage from 600 mV to 100 mV which result in reduction of percentage of missed events from 36 % to 24 % (see Figures S9a and S9b).

To further investigate the origin of missed events, we performed an additional control experiment using the same fluidic cell but a different amplifier, Chimera VC400, with a substantially higher bandwidth, 5MHz. Since a higher bandwidth improves the temporal resolution of the measurement, it is expected to reduce the number of undetected fast translocation events. Consistent with this expectation, we observed a significant reduction in the percentage of missed events to less than 3% when using the higher-bandwidth amplifier (see Figures S9c and S9d). Hence, these results indicate that limited temporal resolution is the dominant source of missed events in our measurements. Therefore, while some degree of protein adsorption onto PDMS cannot be completely excluded, its contribution to the overall percentage of missed events is expected to be minor. Consequently, protein adsorption is unlikely to have a significant impact on the concentration measurements reported in this work. It must be noted that the minimum detectable pulse durations changes by changing the bandwidth and in our experiment 35 µs was used to ensure most of the translocation events were fully resolved.

*Figure S9. Dwell time distributions for 1 µM GFP protein translocation under various experimental conditions in flow cell on nanopore with diameter and nominal length of 8 nm and 12 nm, respectively, along with the fitted FPT model, for 600 mV (a) and 100 mV (b) applied bias voltage measured by using Axopatch 200B with bandwidth of 100 kHz. Same sample was also measured on same nanopore using a Chimera system with bandwidth of 5 MHz when applying 600 mV (c) and 100 mV (d) applied bias voltage across nanopore.*

## S9. Device assembly

Figure S10. (a) Exploded diagram of the microfluidic device fabricated by vertically stacking PDMS components and SiN chip via plasma bonding. The top and bottom layers include microfluidic channels for nanopore access, with the top layer embedding a valve microchannel filled with water. Samples, buffers, and Ag/AgCl electrodes are introduced through these layers. The microtrap layer connects the top layer to the nanopore. The via and PDMS gaskets (spin-coated PDMS layers) confine the electric field and compensate for the SiN chip thickness. The orange lines represent the inlet and outlet PEEK tubing, and the blue lines represent the inlets for the valve layer. The red and blue cylinders represent the cis and trans Ag/AgCl electrodes, respectively. (b) An image of actual microfluidic nanopore device along with zoomed in image of the microtrap layer in pressurized and unpressurized valve.

## S10. Role of hydrophilicity in device function

The functionality of the microfluidic device relies on its ability to maintain a nanometer-scale film of electrolyte beneath the microvalve after closure. This thin film ensures that the device can sustain electrical access to the nanopore, which is crucial for its operation. Hence, plasma treatment was used to increase the hydrophilicity of the PDMS and help to maintain electrical access after valve closure. As can be seen in Figure S11, before plasma treatment, the conductance of the system decreases over time after valve closure and voltage adjustment, and drift in baseline was observed, leading to a decrease in the capture rate. However, for the device that underwent plasma treatment, upon valve closure and voltage adjustment, the conductance of the system did not change, meaning that the thin layer of electrolyte underneath of the valve layer maintained the electrical access.

Figure S11. (a) Representative ionic current traces for 500 pM of 2kbp dsDNA samples under a 400 mV bias voltage measured using a nanopore with diameter and length of respectively, 10 ± 0.5 nm and 12 ± 2 nm before and after plasma treatment, when valve is pressurized at 1 psi. (b) Cumulative number of events for a hydrophobic device over time which demonstrates that upon valve closure the capture rate decreases due to the decrease in the conductance of the system.

## S11. Nanopore fabrication, enlargement, and size measurement data

Figure S12. Overview of the nanopore fabrication process using CBD. (a) Voltage and current measurements showing clear ionic current leakage after nanopore fabrication. (b, c) Nanopore enlargement achieved by applying short pulses of moderate transmembrane voltage, following the method developed by Beamish et al^10^. (d) Example of the I-V curve for a 7.6 nm pore with a length of 12 nm, along with the equation used to determine the nanopore size. Measurements were performed in 3.6 M LiCl at pH 8, with a solution conductivity of 16.8 S/m.

References

(1)	Talaga, D. S.; Li, J. Single-Molecule Protein Unfolding in Solid State Nanopores. *J. Am. Chem. Soc.* **2009**, *131* (26), 9287–9297. https://doi.org/10.1021/ja901088b.

(2)	Li, J.; Talaga, D. S. The Distribution of DNA Translocation Times in Solid-State Nanopores. *J. Phys.: Condens. Matter* **2010**, *22* (45), 454129. https://doi.org/10.1088/0953-8984/22/45/454129.

(3)	Plesa, C.; Kowalczyk, S. W.; Zinsmeester, R.; Grosberg, A. Y.; Rabin, Y.; Dekker, C. Fast Translocation of Proteins through Solid State Nanopores. *Nano Lett.* **2013**, *13* (2), 658–663. https://doi.org/10.1021/nl3042678.

(4)	Waduge, P.; Hu, R.; Bandarkar, P.; Yamazaki, H.; Cressiot, B.; Zhao, Q.; Whitford, P. C.; Wanunu, M. Nanopore-Based Measurements of Protein Size, Fluctuations, and Conformational Changes. *ACS Nano* **2017**, *11* (6), 5706–5716. https://doi.org/10.1021/acsnano.7b01212.

(5)	Zwanzig, R. Diffusion in a Rough Potential. *Proc. Natl. Acad. Sci. U.S.A.* **1988**, *85* (7), 2029–2030. https://doi.org/10.1073/pnas.85.7.2029.

(6)	Niedzwiecki, D. J.; Grazul, J.; Movileanu, L. Single-Molecule Observation of Protein Adsorption onto an Inorganic Surface. *J. Am. Chem. Soc.* **2010**, *132* (31), 10816–10822. https://doi.org/10.1021/ja1026858.

(7)	Charron, M.; Briggs, K.; King, S.; Waugh, M.; Tabard-Cossa, V. Precise DNA Concentration Measurements with Nanopores by Controlled Counting. *Anal. Chem.* **2019**, *91* (19), 12228–12237. https://doi.org/10.1021/acs.analchem.9b01900.

(8)	Sohi, A. N.; Beamish, E.; Tabard-Cossa, V.; Godin, M. DNA Capture by Nanopore Sensors under Flow. *Anal. Chem.* **2020**, *92* (12), 8108–8116. https://doi.org/10.1021/acs.analchem.9b05778.

(9)	Mohammadi, A.; Safari, M.; Rahmanian, M. Label-Free Isolation of Circulating Tumor Cells Using Negative Lateral Dielectrophoresis-Assisted Inertial Microfluidics. *Microfluid Nanofluid* **2023**, *27* (8), 53. https://doi.org/10.1007/s10404-023-02662-3.

(10)	Beamish, E.; Kwok, H.; Tabard-Cossa, V.; Godin, M. Precise Control of the Size and Noise of Solid-State Nanopores Using High Electric Fields. *Nanotechnology* **2012**, *23* (40), 405301. https://doi.org/10.1088/0957-4484/23/40/405301.
